# RegCFinder: targeted discovery of genomic subregions with differential read density

**DOI:** 10.1093/bioadv/vbad085

**Published:** 2023-07-04

**Authors:** Elena Weiß, Caroline C Friedel

**Affiliations:** Institute of Informatics, Ludwig-Maximilians-Universität München, Amalienstr. 17, Munich 80333, Germany; Institute of Informatics, Ludwig-Maximilians-Universität München, Amalienstr. 17, Munich 80333, Germany

## Abstract

**Motivation:**

To date, no methods are available for the targeted identification of genomic subregions with differences in sequencing read distributions between two conditions. Existing approaches either only determine absolute read number changes, require predefined subdivisions of input windows or average across multiple genes.

**Results:**

Here, we present RegCFinder, which automatically identifies subregions of input windows with differences in read density between two conditions. For this purpose, the problem is defined as an instance of the all maximum scoring subsequences problem, which can be solved in linear time. Subsequently, statistical significance and differential usage of identified subregions are determined with DEXSeq. RegCFinder allows flexible definition of input windows to target the analysis to any regions of interests, e.g. promoters, gene bodies, peak regions and more. Furthermore, any type of sequencing assay can be used as input; thus, RegCFinder lends itself to a wide range of applications. We illustrate the usefulness of RegCFinder on two applications, where we can both confirm previous results and identify interesting gene subgroups with distinctive changes in read distributions.

**Availability and implementation:**

RegCFinder is implemented as a workflow for the workflow management system Watchdog and available at: https://github.com/watchdog-wms/watchdog-wms-workflows/

**Supplementary information:**

Supplementary data are available at *Bioinformatics Advances* online.

## 1 Introduction

Functional genomics assays using high-throughput sequencing provide unparalleled opportunities for investigating cellular processes at unprecedented detail. To name just two examples, ChIP-seq and similar assays allow genome-wide mapping of DNA–protein interactions, e.g. for transcription factors and histones (Johnson *et al.*, 2007). Precision nuclear run-on analysis (PRO-seq) sequences nascent RNA 3' ends and thus allows studying active RNA Polymerase II (Pol II) transcription in a strand-specific manner (Mahat *et al.*, 2016).

To identify differences between conditions probed with functional genomics assays, numerous computational and statistical methods have been developed. One commonly used approach employs differential analysis methods on read count data, e.g. DESeq2 (Love *et al.*, 2014), to determine log2 fold-changes in read counts and statistical significance for selected genomic windows. These windows can be either user-defined, e.g. windows around the transcription start site (TSS) for investigating promoter-proximal Pol II pausing, or identified using peak calling, e.g. for differential transcription factor binding analysis. This approach is implemented in the Bioconductor package DiffBind (Ross-Innes *et al.*, 2012), which applies DESeq2 or edgeR after identifying a consensus peak set for all samples. This method considers only the total number of reads in each genomic window, but not how the reads are distributed. Thus, a change in the distribution of reads within a window, e.g. due to changes in Pol II pausing or occupancy of DNA binding proteins, without (significant) changes in the total number of reads would not be identified as differential.

To identify read distribution changes between conditions for particular types of genomic windows (e.g. promoters, gene bodies), metagene plots are commonly used. These show the average read distribution profile for sets of genomic regions. While they allow identifying general trends, individual genomic windows can deviate substantially from the general trend and changes affecting only a minority of genomic windows are often missed. Furthermore, observed changes can only be correlated to other properties of individual genomic windows, such as, e.g. gene length or sequence composition, by subdividing windows into subgroups based on these other properties and then performing metagene analyses separately for subgroups. We previously used this approach to show that CDK12 inhibition triggers a Pol II processivity defect preferentially for long, poly(A)-signal-rich genes (Chirackal Manavalan *et al.*, 2019). More recently, we used clustering of read distribution profiles for promoter windows in PRO-seq data for Herpes Simplex virus 1 (HSV-1) infection to identify subsets of genes with different Pol II pausing changes during HSV-1 infection (Weiß *et al.*, 2023). Metagene analyses of clusters showed that HSV-1 infection induced a downstream shift of Pol II pausing for the majority of host genes. While these metagene analyses provided novel insights in the general impact of CDK12 inhibition and HSV-1 infection, respectively, they did not admit more detailed analyses at single-gene level.

For analysis of Pol II pausing at single-gene level, pausing indices (PIs) have been previously established as a standard metric. The PI of a gene is calculated as the ratio of normalized read counts in a window around the TSS (= promoter window) divided by normalized read counts on the gene body excluding the promoter. PIs are easy to calculate and are altered by changes in the distribution of Pol II occupancy around the TSS and on the gene body. However, while a reduction of PIs is commonly considered evidence of a loss or reduction of Pol II pausing, it can also originate from shifts or an extension of the pausing region downstream of the TSS, as observed, e.g. for HSV-1 infection. Thus, PI changes can be easily misinterpreted and results depend on how wide promoter windows are defined.

*De novo* identification of regions with differential use between conditions can be performed with diffReps (Shen *et al.*, 2013), which was developed for identifying differential chromatin modification sites from ChIP-seq data. It first employs a sliding window approach along the complete genome to identify windows with a significant difference in read counts between two conditions and then merges overlapping windows. While diffReps does not require predefined regions of interest as input, it also does not allow targeting the differential analysis to specific genomic windows of interests. Such genomic windows could not only be peak regions but also promoters, gene bodies, enhancer regions or other types of genomic windows depending on the biological question. While these windows are covered by the sliding windows, the position of the sliding windows relative to the start and end of the regions of interest varies. This complicates the comparison between different regions of interest. Furthermore, all sliding windows are included in the differential analysis, increasing the number of statistical tests performed (one per sliding window). Accordingly, more stringent multiple testing correction is required, which reduces the sensitivity of the approach.

Detection of differentially covered genomic windows is also commonly performed when identifying copy number variants (CNVs) based on read depth (RD). However, these approaches generally assume sudden shifts in RD between consecutive genomic windows with different copy numbers (Magi *et al.*, 2017), which is often not observed for differential regions in functional genomics assays.

Here, we present RegCFinder, a novel method for identifying subregions (= **Reg**ions of **C**hange) of input windows with differences in read distributions between two conditions. RegCFinder can be applied to any type of functional genomics assay and any user-defined genomic windows, such as peak regions, promoters, genes, enhancers, etc. RegCFinder proceeds in two steps: First, regions of change are identified for each input window by reducing this problem to the problem of finding all non-overlapping maximal scoring subsequences in a sequence of real numbers. Second, fold-changes in the relative use of these regions and statistical significance of fold-changes are calculated using DEXSeq (Anders *et al.*, 2012). DEXseq was originally developed to determine differential exon usage from exon counts in multiple RNA-seq replicates for different conditions. This identifies exons of a gene whose relative use compared with the other exons of the same gene increases or decreases. By redefining “genes” as input windows and “exons” as identified regions of change with “filler” regions in-between, we can assess whether there is a statistically significant difference in the use of these regions across replicates of two conditions. We evaluate the usefulness of RegCFinder on the tasks of identifying changes in Pol II pausing upon HSV-1 infection and Pol II processivity upon CDK12 inhibition at single-gene level and compare it against diffReps and XCAVATOR, an RD-based approach for CNV detection. This confirmed both general results from our previous studies, but identified subsets of genes with different characteristics not evident from metagene analyses.

## 2 Methods

### 2.1 General idea

The input to RegCFinder is a set of user-defined genomic windows *W* (in BED format) and aligned read data (in BAM format) for two conditions $c_{1}$ and $c_{2}$ with two or more replicates each (samples $s_{11},\ldots ,s_{1k}$ and $s_{21},\ldots ,s_{2k}$ with k≥2 the number of replicates). RegCFinder then first calculates density functions of the read distribution for each condition and each window in the following way: First, the number of reads mapping to each position i∈w for each window w∈W are determined for each sample. This results in values $r_{11}^{w}(i),\ldots ,r_{1k}^{w}(i),r_{21}^{w}(i),\ldots ,r_{2k}^{w}(i)\;\forall i\in w\;\forall w\in W$. Second, read densities $d_{11}^{w},\ldots ,d_{1k}^{w},d_{21}^{w},\ldots ,d_{2k}^{w}$ are calculated for all w∈W and for each condition j∈{1,2} as


(1) $$d_{js}^{w}\left(i\right)=\frac{r_{js}^{w}\left(i\right)}{\sum_{i\in w}r_{js}^{w}\left(i\right)}\text{for }s\in \left[1:k\right],i\in w.$$

Finally, the average density across replicates for each condition is calculated ∀w∈W as


(2) $$d_{j}^{w}\left(i\right)=\frac{1}{k}\sum_{s=1}^{k}d_{js}^{w}\left(i\right)\;\text{for }i\in w.$$

**Figure 1.**
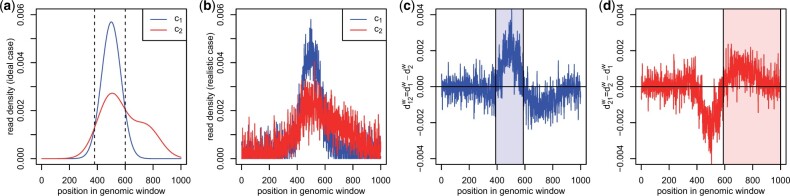
Example illustrating the RegCFinder approach. (**a**) RegCFinder aims to identify regions with differences in read distributions between two conditions. In the ideal case shown here, this can easily be done by identifying the intersection points of the corresponding density functions. (**b**) Sequencing noise, however, leads to noisy read densities ($d_{1}^{w}$ and $d_{2}^{w}$ for condition 1 and 2, respectively) with multiple intersection points. Differences in density functions are calculated from the densities in (b) resulting in sequences $d_{12}^{w}:=d_{1}^{w}-d_{2}^{w}$ (**c**) and $d_{21}^{w}:=d_{2}^{w}-d_{1}^{w}$ (**d**). Maximal scoring subsequences (MSS) are calculated for these sequences and filtered based on randomization (see also Fig. 2). The shaded rectangles mark the final MSS

In the ideal case, densities would be continuous functions as exemplified in Figure 1a but due to noise in the sequencing data, densities are more likely to look as shown in Figure 1b.

The key idea behind RegCFinder is to identify subregions of input windows in which the density is higher in one condition than in the other. In the example in Figure 1a, this would be straightforward as we could simply determine the borders of regions as those positions i∈w for window *w*, where the two density functions cross, i.e. where $d_{1}^{w}(i)=d_{2}^{w}(i)$ (or alternatively where $d_{1}^{w}(i)-d_{2}^{w}(i)=0$, shown as dashed lines in Fig. 1a). However, in the realistic scenario exemplified in Figure 1b, density functions can cross several times splitting the window into many small regions. To prevent this, we want to tolerate some crossovers between densities within a region, provided that the density for one condition tends to be higher than the density for the other condition for most of the region. In terms of the differences between conditions, i.e. $d_{12}^{w}:=d_{1}^{w}-d_{2}^{w}$ (example in Fig. 1c) or $d_{21}^{w}:=d_{2}^{w}-d_{1}^{w}$ (example in Fig. 1d), this means that we aim to identify regions in which either $d_{12}^{w}$ or $d_{21}^{w}$ contains mostly positive values with few negative values in-between (colored rectangles in Fig. 1c and d).

This reduces our problem to a well-known computational problem, i.e. the problem of finding all maximum scoring subsequences (AMSS) in a sequence of real numbers, which can be solved in linear time (Ruzzo and Tompa, 1999). In the following sections, we describe the AMSS problem and how we use the solution to this problem in RegCFinder.

### 2.2 All maximum scoring subsequences

The input to the AMSS problem is a sequence $X=(x_{1},\ldots ,x_{n})$ of real numbers. The score $S_{i,j}$ of a subsequence $\left(x_{i},\ldots ,x_{j}\right)$ of *X* is defined as


(3) $$S_{i,j}=\sum_{l=i}^{j}x_{l}.$$

The score of an empty subsequence is 0.

A maximum scoring subsequence (MSS) of *X* is then defined as a subsequence $m=(x_{i},\ldots ,x_{j})$ of *X* with the following properties:

1. All proper subsequences $m^{\prime }=(x_{k},\ldots ,x_{l})$ of *m* have a lower score than *m*, i.e. $S_{k,l}<S_{i,j}$.
2. No proper supersequence of *m* fulfills property 1.

As the empty sequence is also a subsequence of any subsequence of *X*, $S_{i,j}>0$ for any MSS of *X*. Furthermore, the MSS of *X* are disjoint and every $x_{j}\in X$ with $x_{j}>0$, i.e. every positive element of *X*, is contained in an MSS of *X*. The AMSS problem is simply the problem of finding **all** MSS of *X*. A linear-time algorithm for solving this problem was developed by Ruzzo and Tompa (1999).

### 2.3 Calculation of MSS in RegCFinder

RegCFinder implements the algorithm by Ruzzo and Tompa to separately calculate all MSS for the sequences $d_{12}^{w}=(d_{12}^{w}(1),\ldots ,d_{12}^{w}(|w|))$ and $d_{21}^{w}=(d_{21}^{w}(1),\ldots ,d_{22}^{w}(|w|))$ for each window *w* with two small modifications. First, we multiply each element in the sequence by 100 to obtain larger values and, second, we subtract a small value from each element in the sequence. This serves to prevent long stretches of zeroes in a sequence, which would lead to artificially long MSS if the sequence contains positive elements at the end of these long stretches of zeroes. For instance, for X=(1,0,0,0,0,0,0,0,0,1), the only MSS is the complete sequence.

The final input sequences for window *w*, for which MSS are calculated in RegCFinder, are thus defined as


(4) $$X_{st}^{w}\left(i\right)=100\times d_{st}^{w}\left(i\right)-\frac{\rho }{\left|w\right|}\;\text{for}\;1\leq i\leq \left|w\right|,s,t\in \left\{1,2\right\},s\neq t.$$

Here, ρ is a pseudocount, which is set to 1 by default. Since $\sum_{i=1}^{\left|w\right|}d_{s}^{w}(i)=1$ for s∈{1,2}, we have for s,t∈{1,2},s≠t that


(5) $$\begin{array}{c} \sum_{i=1}^{\left|w\right|}X_{st}^{w}(i)=100\times \sum_{i=1}^{\left|w\right|}d_{st}^{w}(i)-\sum_{i=1}^{\left|w\right|}\frac{\rho }{\left|w\right|}\\ =100\times \left(\sum_{i=1}^{\left|w\right|}d_{s}^{w}(i)-\sum_{i=1}^{\left|w\right|}d_{t}^{w}(i)\right)-\rho =-\rho . \end{array}$$

As a consequence, long MSS are penalized with a linear penality function $p\left(\lambda \right)=\frac{\rho }{\left|w\right|}\times \lambda$, where λ is the length of the MSS. Thus, higher values of ρ lead to shorter MSS.

### 2.4 Filtering and merging of MSS

**Figure 2.**
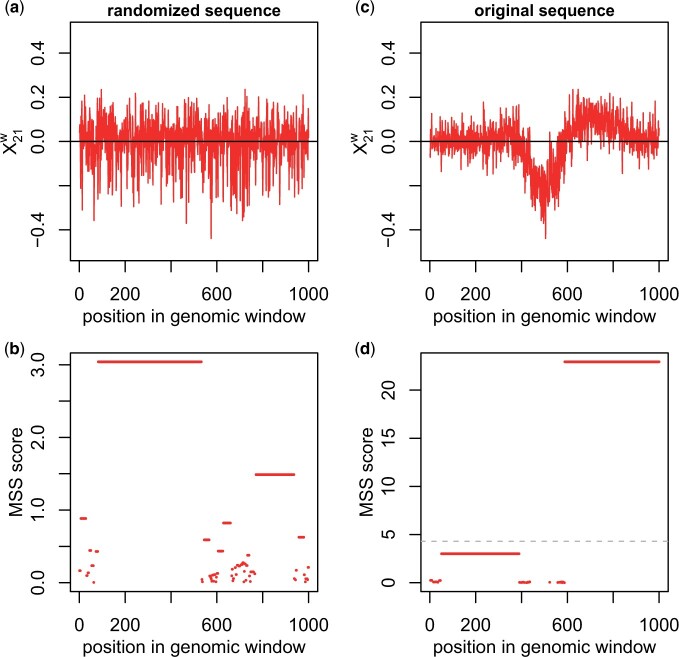
(**a**) Randomized and (**c**) original input sequence obtained from the example in Figure 1b and d. (**b**, **d**) MSS identified on the randomized (b) and original (d) sequence. Each MSS is shown as a horizontal line covering the positions in the genomic window shown on the *x*-axis. The *y*-axis position of the MSS indicates its score. The dashed line in (d) shows the maximum MSS score across 10 randomizations. All MSS with a score less or equal to this score are discarded, resulting in the identification of the one region of change shown in Figure 1d

In the following, let $M_{12}$ be the set of MSS determined for $X_{12}^{w}$ and $M_{21}$ be the set of MSS for $X_{21}^{w}$. As noted above, any positive element of the input sequence *X* is contained in an MSS. Thus, even without long stretches of mostly positive values, MSS will be identified. In the worst case, every positive element will be an MSS of length 1, e.g. as for the sequence X=(1,−2,1,−2,1,−2,1) (MSS underlined). Furthermore, even random sequences can contain long MSS if they contain a sufficient number of large positive elements larger than absolute values of most negative values (see Fig. 2a and b).

To identify and remove such MSS that are no better than random, we repeatedly randomly permute each of the input sequences *X* by sampling |X| times from *X* without replacement (default = 1000 randomizations). We then identify MSS for the randomized sequences and remove all MSS for the original sequence with a score less than or equal to the maximum MSS score identified for any of the randomized sequences. In case of the example shown in Figure 2c (calculated from the example in Fig. 1d), all identified MSS are removed with a score below the gray dotted line in Figure 2d. Thus, for the example in Figure 1 only one region would be identified in which the density for condition 2 is higher than the density of condition 1 (red transparent rectangle in Fig. 1d). In this way, RegCFinder also filters regions with only small differences in the distributions (e.g. the region left of the left dashed vertical line in Fig. 1a).

After filtering MSS, $M_{12}$ and $M_{21}$ are merged to obtain the final regions of change. In case of overlapping MSS $m_{12}\in M_{12}$ and $m_{21}\in M_{21}$, only the MSS with the highest score is retained. Regions with higher density in condition 1 ($c_{1}$) than in condition 2 ($c_{2}$) are denoted as $c_{1}>c_{2}$ regions and regions with higher density in condition 2 than in condition 1 are denoted as $c_{2}>c_{1}$ regions.

### 2.5 Significance assessment using DEXSeq

To assess statistical significance of differential and log2 fold-changes in relative use of the identified regions given the number of reads in each replicate, RegCFinder uses DEXSeq (Anders *et al.*, 2012). For this purpose, a new annotation file is generated for DEXSeq with “genes” defined as the input windows. The “exons” consist of the identified regions of change and “filler” regions representing the genomic regions within the window between and around regions of change. For the example in Figure 1, five exons would be defined: two regions of change (blue and red rectangle in Figure 1c and d, respectively) and three filler regions (i) on the left of the first region, (ii) on the right of the second region and (iii) between the two regions. Read counts for exons are determined using featureCounts (Liao *et al.*, 2014) on input BAM files. DEXSeq results are included in the final output file (in TSV format), which contains coordinates of the identified regions, their MSS score and their log2 fold-change and multiple testing adjusted *P*-value from DEXSeq.

## 3 Results

### 3.1 Input data

We evaluated RegCFinder on two datasets, which we previously investigated using metagene analyses to identify differences in Pol II pausing (Weiß *et al.*, 2023) and Pol II processivity (Chirackal Manavalan *et al.*, 2019), respectively. The first dataset was obtained with PRO-seq for mock infection and 3 h post wild-type (WT) HSV-1 infection (three replicates each) by Birkenheuer *et al.* (2018). Here, mock infection means that cells were exposed to the same medium as the HSV-1-infected cells that lacked virus. We previously used clustering of read distributions around the TSS and metagene analyses of clusters to show that WT HSV-1 infection leads to a downstream shift in Pol II pausing for most host genes (Weiß *et al.*, 2023). Unfortunately, the metagene analysis did not allow further investigating this effect at single-gene level. PI analysis on this dataset showed widespread reductions in PIs for most genes, which can be misinterpreted as a loss in Pol II pausing and increased elongation.

The second dataset consisted of ChIP-seq data for Pol II and Ser2 phosphorylations (P-Ser2) of the Pol II carboxy-terminal domain (CTD) in a cell line expressing an analog-sensitive version of the CDK12 kinase (Chirackal Manavalan *et al.*, 2019). This analog-sensitive CDK12 is inhibited by the ATP analog 3-MB-PP1. Three replicates were obtained each with either DMSO (Ctl) or 3-MB-PP1 (Inhi) treatment for 4.5 h. We previously reported that CDK12 inhibition induces a Pol II processivity defect characterized by a loss of ChIP-seq read coverage toward 3' ends of predominantly long, poly(A)-signal-rich genes and a shift of the terminal P-Ser2 peak into the gene body (Chirackal Manavalan *et al.*, 2019) (see Supplementary Fig. S7a for an example). Matching RNA-seq of nuclear RNA showed that this was associated with premature transcription termination at poly(A) signals within gene bodies.

Data preprocessing is described in the Supplementary Material.

### 3.2 Changes in Pol II pausing during HSV-1 infection

We first applied RegCFinder to the PRO-seq samples of mock and WT HSV-1 infection for promoter windows defined as ±3 kb around the TSS of 7650 genes (with the default of 1000 randomizations). These TSS were previously identified from PROcap-seq and PRO-seq data of flavopiridol-treated uninfected human foreskin fibroblasts (Weiß *et al.*, 2023) (see Supplementary Material). PROcap-seq is a variation of PRO-seq that specifically maps Pol II initiation sites. Flavopiridol inhibits CDK9, which is required for the switch to active elongation, and arrests Pol II in a paused state at the promoter. This allows also identifying the TSS for genes that are not or weakly paused in untreated cells.

RegCFinder identified a total of 7621 regions of change for the input windows. For 7201 regions, a *P*-value was calculated by DEXSeq. The remaining 420 regions were excluded by DEXSeq in the independent filtering step, which excludes regions with low read counts. For 6958 regions (96.7% of regions with *P*-values), a significant change was observed (multiple testing adjusted *P*-value ≤0.01). For comparison, a test with only 10 randomizations instead of the default 1000 randomizations identified 10 528 regions of change for which DEXSeq calculated *P*-values, but only 88% of these were statistically significant (adj. *P* ≤ 0.01). Thus, by increasing the number of randomizations, significance of results can be improved at the cost of reduced sensitivity.

Of the 7201 regions of change with *P*-values, 3414 had a higher density in mock than WT (mock>WT regions) and 3787 had a higher density in WT (WT>mock regions). Fold-changes determined by DEXseq were consistent with the RegCFinder predictions as mock>WT regions had positive log2 fold-changes in the comparison of mock versus WT and WT>mock regions had negative log2 fold-changes (Supplementary Fig. S1). In contrast, the median log2 fold-change of the 10 072 filler regions was close to zero and only 1697 filler regions (17%) showed a statistically significant change. Notably, with 10 randomizations, only 8% of the filler regions were statistically significant. Since more randomizations lead to more stringent filtering, some correct regions of change identified with 10 randomizations are thus filtered with 1000 randomizations and instead included as filler regions.

**Figure 3.**
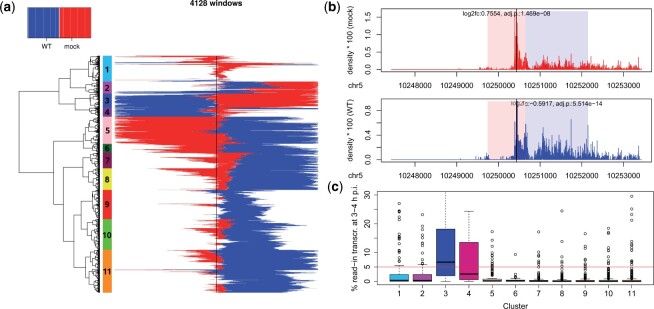
(**a**) Heatmap showing the location and type of identified regions of change (red = mock>WT, blue = WT>mock) for 4128 windows with at least one region with *P*-values determined by DEXSeq. For details, see the main text. The TSS is indicated by a black vertical line. (**b**) Read density in mock (red, top panel) and WT (blue, bottom panel) infection for an example from cluster 10. Windows are shown in 5'–3' direction, i.e. regions up- and downstream of the TSS are to the left and right, respectively, of the TSS (black vertical line). Red shaded rectangle = mock>WT region, blue shaded rectangle = WT>mock regions. Log2 fold-changes and adj. *P*-values in mock versus WT infection are shown in the top panel for mock>WT regions and in the bottom panel for WT>mock regions. (**c**) Boxplots showing the distribution of % read-in transcription (for definition, see the text) at 3–4 h p.i. for the 11 clusters marked in (a). The red horizontal line indicates the cutoff (5%) we previously used for identifying genes with read-in transcription

Figure 3a visualizes the location of the identified regions within the input promoter windows for the 4128 windows containing at least one region with a *P*-value from DEXseq. Here, each row represents one window and each column a position in the window (from −3 kb upstream of the TSS to +3 kb downstream of the TSS, red = mock>WT regions, blue = WT>mock regions, white = filler regions or region without DEXseq *P*-value). Windows were clustered according to Euclidean distances and Ward’s clustering criterion. A cutoff on the clustering dendogram was chosen manually to obtain the 11 clusters marked in Figure 3a. Supplementary Figure S2 shows log2 fold-changes in mock versus WT determined with DEXseq for regions with adj. *P* ≤ 0.01 for windows ordered as in Figure 3a. The latter confirms the high consistency between log2 fold-changes determined by DEXseq and the type of region determined by RegCFinder.

Figure 3a reveals both interesting subgroups as well as general trends. With some exceptions (clusters 1–4), WT>mock regions extended downstream of the TSS, while mock>WT regions were located around or upstream of the TSS. However, there were strong differences with regard to how far downstream of the TSS the WT>mock regions extended. For clusters 9, 10 and parts of cluster 11, the WT>mock regions ended well before the 3' end of the promoter windows. This is consistent with a downstream shift of Pol II pausing and confirmed by inspection of read distributions for individual genes (Fig. 3b) and metagene analyses (Supplementary Fig. S3a). For other clusters (5–8, partly 11), WT>mock regions extended to or close to the 3' end of promoter windows suggesting either a further downstream shift of pause sites or increased elongation due to a loss of pausing. While the metagene analyses suggest the former (Supplementary Fig. S3b and c), inspection of individual genes identifies both examples for increased elongation (Supplementary Fig. S4a) and increased (relative) use of downstream pause sites (Supplementary Fig. S4b). This reflects the limits of metagene analyses due to averaging across genes with potentially diverse patterns.

Interestingly, clusters 3 and 4 had long WT>mock regions upstream of the TSS. We previously showed using 4sU-seq that HSV-1 infection disrupts transcription termination, leading to extensive read-through transcription beyond poly(A) sites that can extend for tens-of-thousands of nucleotides into intergenic regions and into downstream genes (Rutkowski *et al.*, 2015). 4sU-seq is based on labeling newly transcribed RNA with 4-thiouridine (4sU) in specific time intervals (here: 1 h intervals during infection) followed by sequencing of labeled RNA. Read-through transcription extending into a downstream gene is denoted as “read-in transcription” and calculated as expression in the 5-kb upstream of the gene start divided by gene expression (Hennig *et al.*, 2018). [Formal definition: % read-in transcription = 100 × fragments per million mapped reads (FPKM) in the 5 kb upstream of the gene start divided by the gene FPKM. Values for uninfected cells are subtracted from values for infected cells and negative values are set to 0]. Analysis of read-in transcription previously determined using 4sU-seq for mock and 3–4 h p.i. HSV-1 infection (Rutkowski *et al.*, 2015) showed significant read-in transcription for clusters 3 and 4 (Fig. 3c, metagene plots in Supplementary Fig. S3d and e). Thus, WT>mock regions identified by RegCFinder upstream of the TSS reflect read-in transcription for these genes.

### 3.3 Impact of CDK12 inhibition on Pol II processivity

As a second analysis, we applied RegCFinder to ChIP-seq data for Pol II and P-Ser2 with DMSO (Ctl) or 3-MB-PP1 (Inhi) treatment for 4.5 h. Here, we used windows covering complete genes from −3 kb upstream of the TSS to +3 kb downstream of the transcription termination site (TTS). We included only genes with a distance ≥5 kb to the next up- and downstream gene (=8086 gene windows).

More regions of change were identified for P-Ser2 (10 218 with *P*-values calculated by DEXseq) than for Pol II (7065) and a larger fraction of P-Ser2 regions were significant (90%) than of Pol II regions (83%). This can be explained by the fact that Pol II ChIP-seq reads are often concentrated in the promoter region due to Pol II pausing, resulting in lower coverage on gene bodies (Yu *et al.*, 2015). In contrast, P-Ser2 reads more evenly cover the gene body with a less prominent peak shortly downstream of the TTS (see e.g. Supplementary Fig. S7a). Again, log2 fold-changes determined by DEXSeq were consistent with the direction of change identified by RegCFinder (Supplementary Fig. S5).

**Figure 4.**
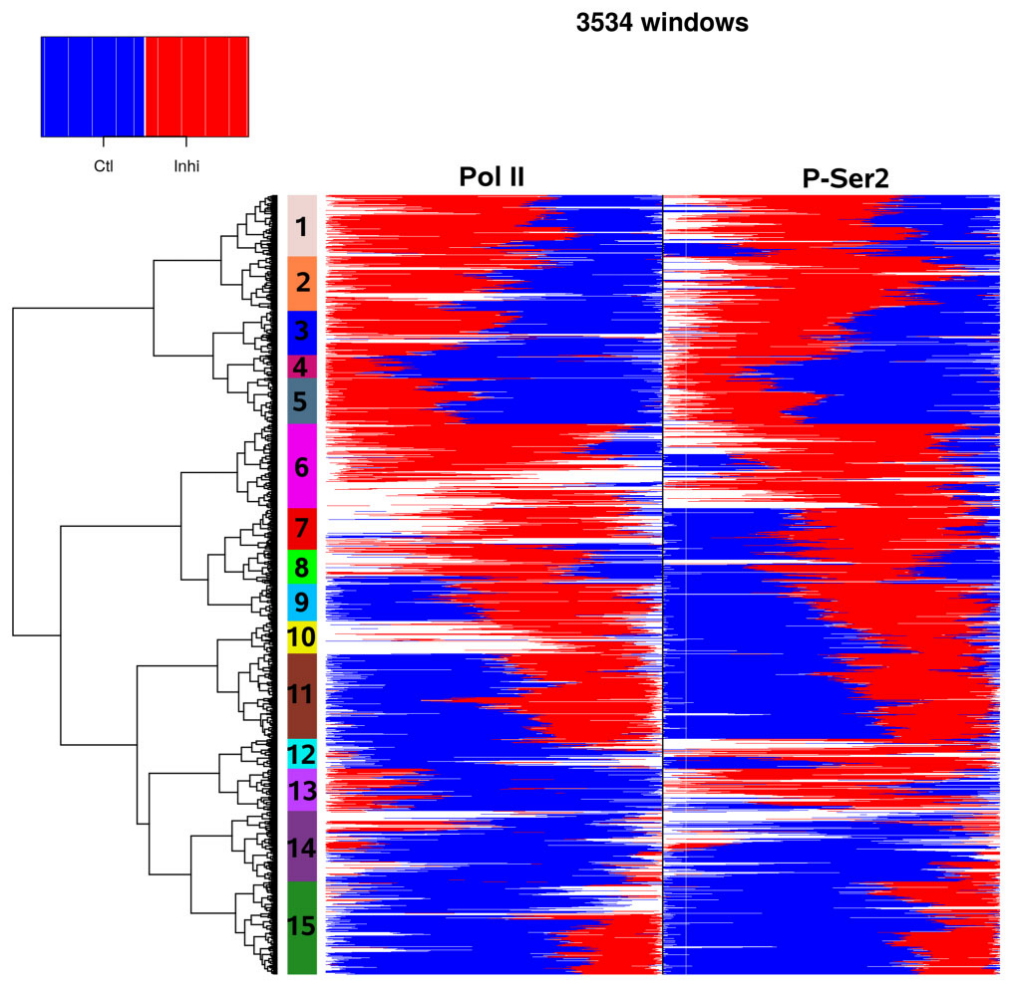
Heatmap showing the location of identified regions of change in Pol II and P-Ser2 ChIP-seq data for control (Ctl) and CDK12 inhibitor (Inhi) treatment. Location is shown relative to the window start and end. Inhi>Ctl regions are indicated in red and Ctl>Inhi regions are indicated in blue. Filler regions and regions without a *P*-value calculated by DEXSeq are shown in white. For more details, see the main text

For 3405 and 4639 genes at least one statistically significant region of change was identified for Pol II and P-Ser2, respectively. Here, 74% and 86%, respectively, of these genes contained both a significant Inhi>Ctl and Ctl>Inhi region. The heatmap in Figure 4 visualizes the identified regions of change in Pol II and P-Ser2 for the 3534 genes for which at least one region of change was identified in both Pol II and P-Ser2 similar to Figure 3. Here, the positions of the identified regions relative to the 5' (left) and 3' end of the window (right) are shown. Regions were clustered as described above and 15 clusters were obtained from the clustering dendogram (marked in Fig. 4). Results were generally highly consistent between Pol II and P-Ser2 ChIP-seq, with regions of changes of the same type identified at similar positions.

Clusters 1–5 exhibited the pattern we expected from our previous metagene analysis, which showed a loss of Pol II from gene 3' ends and a shift of 3' end P-Ser2 peaks into the gene body upon CDK12 inhibition (Chirackal Manavalan *et al.*, 2019). Accordingly, Inhi>Ctl regions (red), i.e. regions with a relative increase upon inhibitor treatment, were located closer to the TSS and Ctl>Inhi regions (blue) were located closer to the TTS. An example gene for cluster 1 is shown in Supplementary Figure S7a. In this case, the Inhi>Ctl region found for P-Ser2 approximately matched the terminal P-Ser2 peak shifted upstream upon CDK12 inhibition.

**Figure 5.**
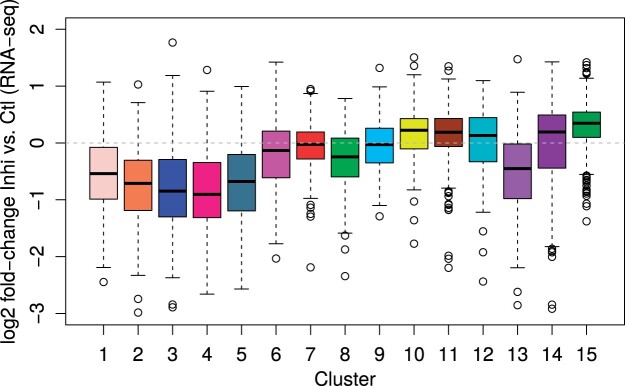
Boxplots showing the distribution of log2 fold-changes in nuclear RNA between CDK12 inhibitor and control treatment for the clusters from Figure 4

Previously, we found that longer genes were more strongly affected by CDK12 inhibition and loss of Pol II at gene 3' ends resulted in a reduction in nuclear RNA levels for corresponding genes (Chirackal Manavalan *et al.*, 2019). As illustrated in the example gene in Supplementary Figure S7a, this is not due to down-regulation of the complete gene, but rather due to premature transcription termination leading to a loss of reads in the 3' end region of the gene. Consistent with this, clusters 4 and 5, for which Inhi>Ctl regions ended relatively close to the gene start, indicating a strong shift of Pol II from the gene 3' end toward the gene 5' end, contained longest genes (Supplementary Fig. S6). Moreover, clusters 1–5 all showed a strong reduction in nuclear RNA levels upon CDK12 inhibition and stronger reduction was observed for clusters with Inhi>Ctl regions ending closer to the TSS (Fig. 5).

For cluster 6, Inhi>Ctl regions in Pol II and P-Ser2 cover a large fraction of the gene and are followed by only short or no Ctl>Inhi regions at the 3' end. Thus, the Pol II processivity defect is only noticeable close to the gene 3' end. Consistently, these genes were short and little reduction in nuclear RNA was observed. In contrast, cluster 8 showed similar patterns as observed for the example in Supplementary Figure S7a, i.e. an Inhi>Ctl region between two Ctl>Inhi regions for P-Ser2. This central Inhi>Ctl region reflects the shift of the terminal P-Ser2 peak into the gene body. Cluster 13 represents the one example with strong divergence between the Pol II and P-Ser2 results. Manual inspection of example genes showed generally low read coverage on gene bodies, in particular for Pol II, explaining the low consistency between Pol II and P-Ser2 results.

The remaining clusters (clusters 7, 9–11, 14 and 15) showed Inhi>Ctl regions downstream of relatively long Ctl>Inhi regions in either P-Ser2 alone or in both Pol II and P-Ser2. The Inhi>Ctl regions were not followed by additional downstream Ctl>Inhi regions or only very short ones. Notably, genes in these clusters did not show reduced expression upon CDK12 inhibition, with some even showing an increase in expression. An example gene from cluster 15 is shown in Supplementary Figure S7b. Here, no premature transcription termination is observed and this gene is even weakly up-regulated in nuclear RNA (log2 fold-change 0.36, adj. *P* = $6.69\times 10^{-6}$). The Inhi>Ctl region identified by RegCFinder is located upstream of the terminal P-Ser2 peak. While the summit position of the P-Ser2 peak is unchanged, read distributions upstream of this peak clearly differ between control and CDK12 inhibition.

In summary, RegCFinder identified interesting subsets of genes that are not fully explained by our existing model of the effects of CK12 inhibition on Pol II processivity developed based on metagene analyses. One such subset is cluster 2, which shows a strong shift of the terminal P-Ser2 peak into the gene body that cannot be explained by gene length alone. Moreover, 46% of genes shown in Figure 4 (i.e. clusters 7, 9–11, 14 and 15) showed changes in Pol II occupancy upon CDK12 inhibition that do not appear to be directly linked to premature transcription termination and warrant further research.

### 3.4 Comparison to competing approaches

We compared RegCFinder against two alternative approaches that focus on identifying differentially covered regions in the genome: XCAVATOR, a RD-based approach for CNV detection (Magi *et al.*, 2017), and diffReps, developed for detecting differential chromatin modification sites (Shen *et al.*, 2013). Full details of the comparison can be found in the Supplementary Material. In brief, XCAVATOR did not identify any differential regions on the PRO-seq and ChIP-seq data, likely due to the absence of sudden shifts in read coverage. diffReps mostly identified regions with absolute changes in RD. Consequently, results on the PRO-seq data differed strongly. On the ChIP-seq data, diffReps identified a subset of regions identified by RegCFinder on input genes.

## 4 Discussion

In this article, we present RegCFinder, a new approach for determining differences between two conditions in sequencing data. In contrast to previous approaches, it focuses on identifying differences in the distribution of reads at single-gene, or rather single-window, level. Given a set of input windows defined by the user, RegCFinder identifies subregions of these input windows, the so-called regions of change, in which one condition has a higher read density than the other condition. For this purpose, the problem is defined as an instance of the AMSS problem, which can be solved efficiently in linear time (Ruzzo and Tompa, 1999).

Since this problem definition considers only the distribution of reads within the input windows but not the absolute read numbers, statistical significance and log2 fold-changes in the relative use of each identified region of change compared with the rest of this input window are calculated with DEXSeq from read counts. Since we also include “filler” regions that are not part of any identified regions of change in the input for DEXSeq, this step is not limited to identifying regions of change as statistically significant. Indeed, in the two applications shown in this article, 8–17% of filler regions showed a statistically significant change. Nevertheless, this is much lower than the 84–96% of the regions of change identified by RegCFinder that were statistically significant. Furthermore, we observed a trade-off between sensitivity and specificity of RegCFinder that can be tuned by adjusting the number of randomizations used for filtering the identified MSS. Fewer randomizations result in less stringent filtering while more randomizations lead to filtering of some of the truly differential regions, which will then be included as filler regions. Notably, for significance analysis, DEXSeq can be replaced with any other method with a similar purpose.

Since RegCFinder is both agnostic to how input windows are defined by the user and what type of sequencing data is provided as input, it lends itself to a wide range of applications. Here, we illustrated the usefulness of RegCFinder on two applications: (i) Pol II pausing changes upon WT HSV-1 infection analyzed using PRO-seq data and (ii) changes in Pol II processivity upon CDK12 inhibition analyzed using ChIP-seq of Pol II and P-Ser2. In both cases, we previously used metagene analyses on subgroups of genes either defined (i) by clustering of PRO-seq read distributions for mock and WT infection (Weiß *et al.*, 2023) or (ii) based on gene length or differential gene expression (Chirackal Manavalan *et al.*, 2019). While the metagene analyses already yielded interesting novel insights, we were frustrated by their limitations regarding the analysis of individual genes, which motivated the development of RegCFinder. Other approaches, in particular PIs, were also unsatisfactory as they could not distinguish between “normal” Pol II pausing changes with increased elongation and the downstream shifts of pause sites in WT HSV-1 infection.

The analysis of PRO-seq data of mock and WT HSV-1 infection confirmed the downstream shift of Pol II pausing to less well-defined downstream pause sites for a large fraction of genes (clusters 9, 10 and partly 11 in Fig. 3). For other genes (clusters 5–8 and partly 11), increased read density in WT infection downstream of the TSS extended until or close to the end of the promoter window. An investigation of example genes suggested that at least some of these genes may not actually exhibit delayed Pol II pausing but rather increased elongation on the whole gene body. Thus, RegCFinder now allows more detailed analyses of these genes and their characteristics compared with other genes for which pausing is retained at downstream sites. Finally, RegCFinder also identified genes with read-in transcription originating from disrupted transcription termination of an upstream gene. Thus, no prior filtering of these genes was necessary.

Similarly, the analysis of Pol II and P-Ser2 ChIP-seq upon CDK12 inhibition confirmed our previous observations for a large fraction of genes. However, we also identified a large number of genes with different patterns of changes in the Pol II and P-Ser2 distribution that open up new avenues of investigation into the role of CDK12 not evident from the metagene analyses.

RegCFinder also provides new possibilities for integrating different data types. First, location of identified regions of change for two or more types of data (e.g. different ChIP-seq antibodies, different sequencing assays) or different experiments can be easily compared with the heatmap approach shown in Figure 4. Second, the new DEXSeq annotation created from identified regions of change for one data type or experiment can be directly used to calculate differential use on a different data type or in a different experiment. For instance, one could analyze if P-Ser2 regions showed the same differential use in Pol II ChIP-seq data or ChIP-seq data for other CTD phosphorylations, elongation factors, histone modifications or in nuclear RNA-seq data.

The limitations of RegCFinder should also be noted. First, it is designed for pairwise comparisons of conditions. Thus, it cannot be used for segmentation of input windows given only one condition and comparison of three or more conditions requires comparison to a common reference (similar to other differential approaches like differential gene expression or exon usage). Second, depending on the noise level in the data, the precise border positions of regions of change may be difficult to determine using the RegCFinder approach and thus will likely vary between biological replicates or different RegCFinder parameter settings. In cases in which borders can be more accurately determined by other means, e.g. reads crossing splice junctions in case of RNA-seq, it may thus be better to use these other means or complement the RegCFinder results with such other information. Finally, RegCFinder is targeted toward applications in which the read distribution provides information, such as functional genomics approaches based on short read sequencing, like ChIP-seq, PRO-seq, ATAC-seq and more. While in principle RegCFinder could also be applied to read densities obtained from long-read sequencing, the applications usually addressed by long-read sequencing likely are not suited for the RegCFinder approach.

In summary, RegCFinder implements a novel approach for identifying genomic regions with differences in read density between two conditions. Due to its flexibility regarding the definition of input windows and the type of input sequencing data, we believe it will be of broad use for a wide range of biological questions.

## Supplementary Material

vbad085_Supplementary_Data

## Data Availability

RegCFinder was implemented as a workflow for the workflow management system Watchdog (Kluge and Friedel, 2018; Kluge *et al.*, 2020) and uses Conda for automatic software deployment (https://docs.conda.io). The RegCFinder workflow is available in the Watchdog workflow repository at https://github.com/watchdog-wms/watchdog-wms-workflows together with a detailed README file on installing and running the workflow (including installation instructions for Watchdog) and in- and output file formats. All modules used in this workflow, including pre-existing modules (featureCounts, mergeFeatureCounts and DEXSeq) and modules newly developed for RegCFinder (amss, quantCurveScore, both implemented in R, preDexseq, implemented as a bash script) are available in the Watchdog module repository at https://github.com/watchdog-wms/watchdog-wms-modules. Download links and more detailed documentation for Watchdog can be found at https://www.bio.ifi.lmu.de/watchdog. The data underlying this article are available in Gene Expression Omnibus at https://www.ncbi.nlm.nih.gov/geo/ and can be accessed with accession GSE106126 (PRO-seq data for mock and HSV-1 infection) and accession GSE120072 (ChIP-seq data of CDK12 inhibition).
