# Supplementary material for: RegCFinder: targeted discovery of genomic subregions with differential read density
Source: Bioinform Adv. 2023 Jul 4;3(1):vbad085. doi: 10.1093/bioadv/vbad085 (PMC10343947; doi:10.1093/bioadv/vbad085)
Supplement: vbad085_Supplementary_Data [file vbad085_supplementary_data.pdf]

RegCFinder: targeted discovery of genomic subregions with differential read density
Supplementary Material

Elena Weiß and Caroline C. Friedel

1 Data preprocessing

PRO-seq data were aligned against the human genome (GRCh37/hg19), human rRNA sequences, and the HSV-1 genome (GenBank accession code: JN555585) using ContextMap2 version 2.7.9 [Bonfert et al., 2015] (using BWA [Li and Durbin, 2009] as short read aligner and allowing a maximum indel size of 3 and at most 5 mismatches). For the two repeat regions in the HSV-1 genome, only one copy was retained each, excluding nucleotides 1–9,213 and 145,590–152,222 from the alignment. ChIP-seq reads were aligned to the human genome (hGRCh38/g38) using BWA [Li and Durbin, 2009]. Reads with an alignment score < 20 were discarded. SAM output files of aligners were converted to BAM files using samtools [Danecek et al., 2021].

Log2 fold-changes in nuclear RNA for CDK12 inhibition vs. control were taken from our previous publication [Chirackal Manavalan et al., 2019].

2 Identification of TSS

To identify TSS from the PROcap-seq and PRO-seq of flavopiridol-treated cells, the iTiSS program [Jürges et al., 2021] was run separately for each sample in the SPARSE_PEAK mode with standard parameters. Afterward, the iTiSS TSRMerger program was used to select only peaks that were identified in both samples within ± 5 bp resulting in 136,090 putative TSS positions. These were further filtered by requiring a maximum distance of 500 bp to the nearest annotated gene. This resulted in 42,193 potential TSS positions for 7,650 genes. The TSS with the highest expression was selected for each gene.

3 Supplementary Figures

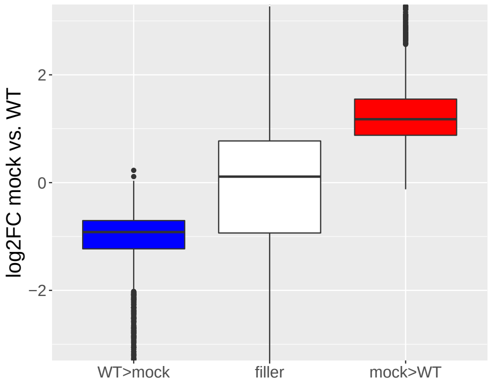

Supplementary Fig. S1 Boxplot showing log2 fold-changes for mock vs. WT infection determined with DEXseq for WT>mock, mock>WT regions and filler regions. Positive log2 fold-changes indicate increased use in mock and negative log2 fold-changes increased use in WT.

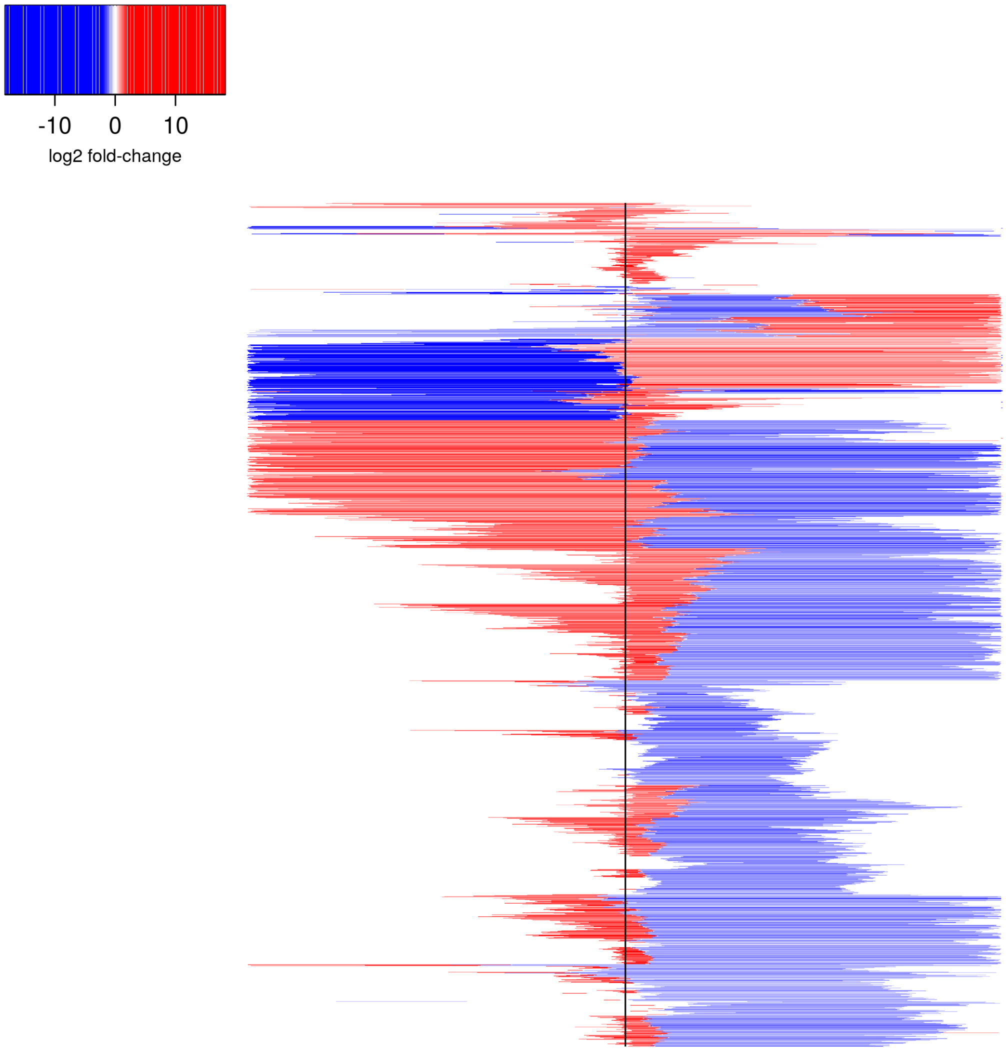

Supplementary Fig. S2 Heatmap illustrating log2 fold-changes in mock vs. WT determined with DEXseq for regions of change with adj. $p \leq 0.01$. The order of windows is the same as in Fig. 3a. Here, all regions with log2 fold-change ≤ -2 or log2 fold-change ≥ 2 are shown with the darkest blue or red, respectively. Filler regions and regions with no p-value or adj. $p > 0.01$ are shown in white.

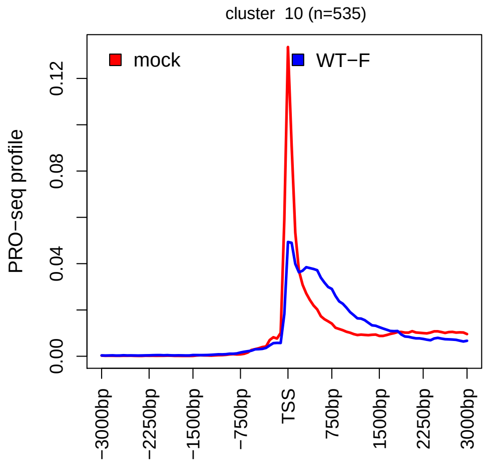

(a)

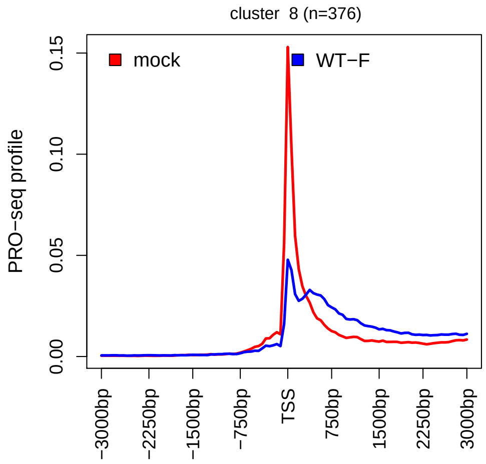

(b)

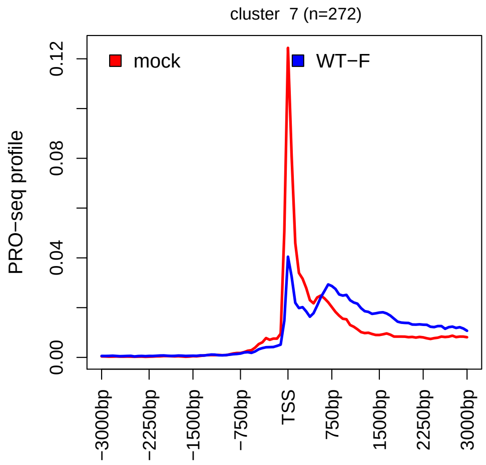

(c)

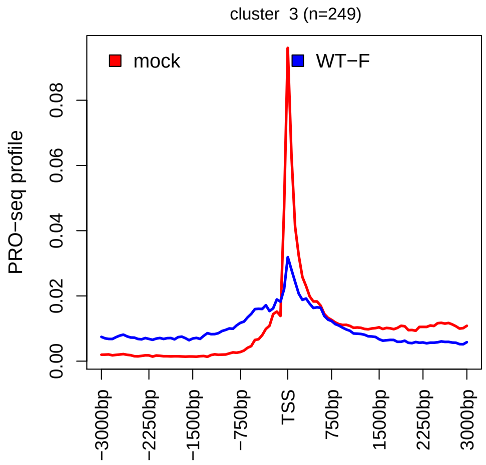

(d)

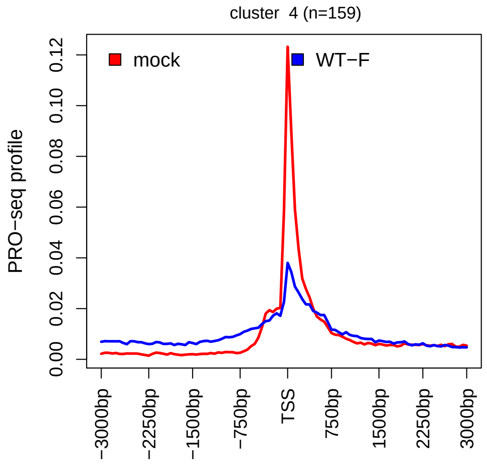

(e)

Supplementary Fig. S3 Metagene curves in the $\pm$ 3 kb around the TSS for mock (red) and WT (blue) infection for selected clusters from Fig. 3 in the main manuscript (cluster numbers and number of genes in each cluster on top of subfigures). For this purpose, the regions -3 kb to +3 kb of the TSS were divided into 101 bp bins for each gene. For each bin, the average coverage per genome position was calculated in a strand-specific manner for PRO-seq data and bin read coverages were then normalized by dividing by the total sum of all bins. Metagene curves for each replicate were created by averaging results for corresponding bins across all genes and then showing the average metagene curves across replicates.

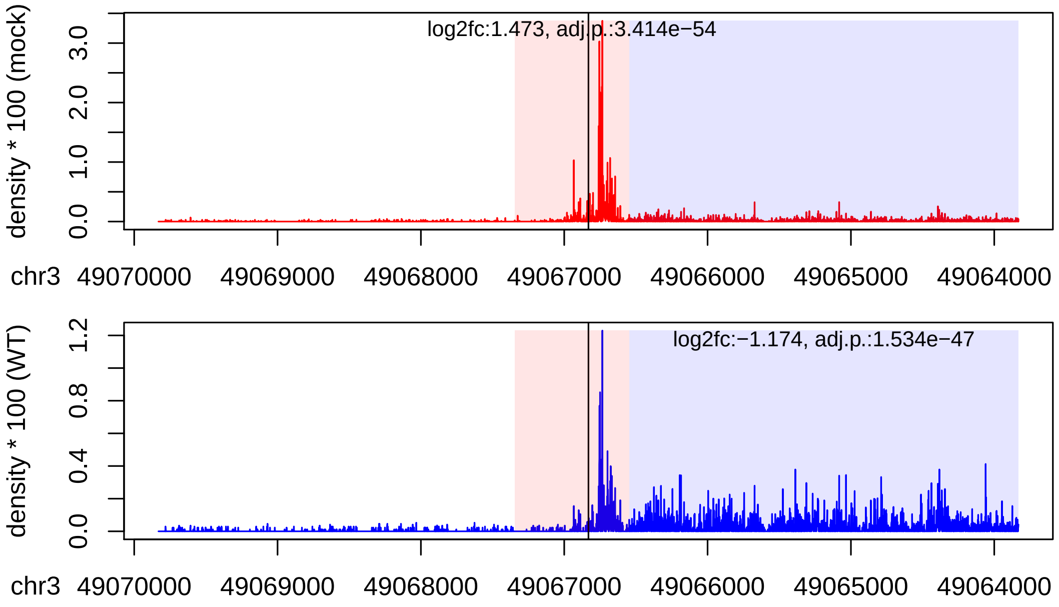

(a)

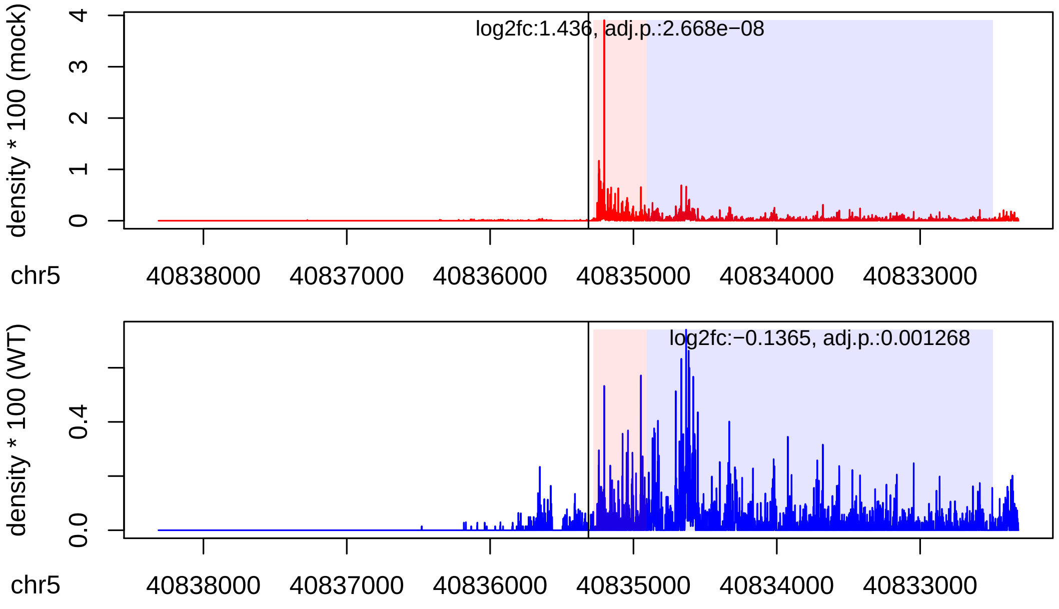

(b)

Supplementary Fig. S4 Read density in mock (red, top panel) and WT (blue, bottom panel) infection for example windows from cluster 8. The black vertical line marks the TSS and windows are shown in 5' to 3' direction, i.e. regions up- and downstream of the TSS are to the left and right, respectively, of the vertical line. mock>WT regions are marked by a red shaded rectangle and WT>mock regions by a blue shaded rectangle. Log2 fold-changes and adj. p-values in mock vs. WT infection are shown in the top panel for mock>WT regions and in the bottom panel for WT>mock regions.

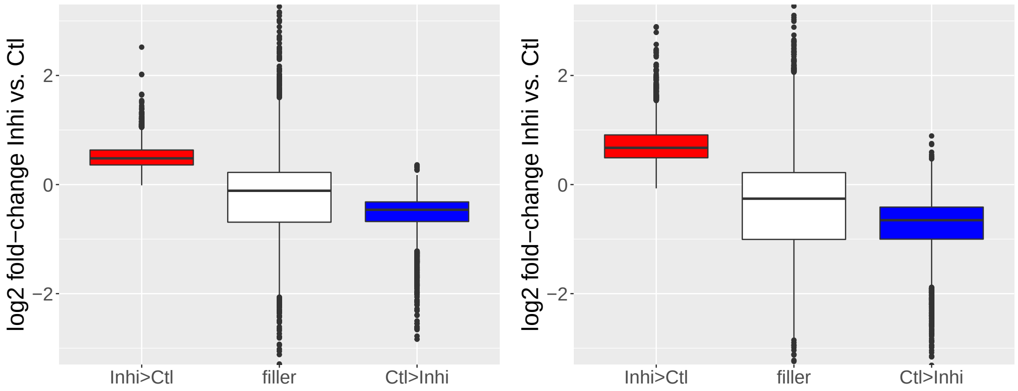

(a) **(b)**

Supplementary Fig. S5 Boxplots showing log2 fold-changes for CDK12 inhibitor treatment (Inhi) vs. control (Ctl) determined with DEXseq for Inhi>Ctl, Ctl>Inhi and filler regions determined from Pol II (a) and P-Ser2 ChIP-seq (b). Positive log2 fold-changes indicate higher use in Inhi and negative log2 fold-changes higher use in Ctl.

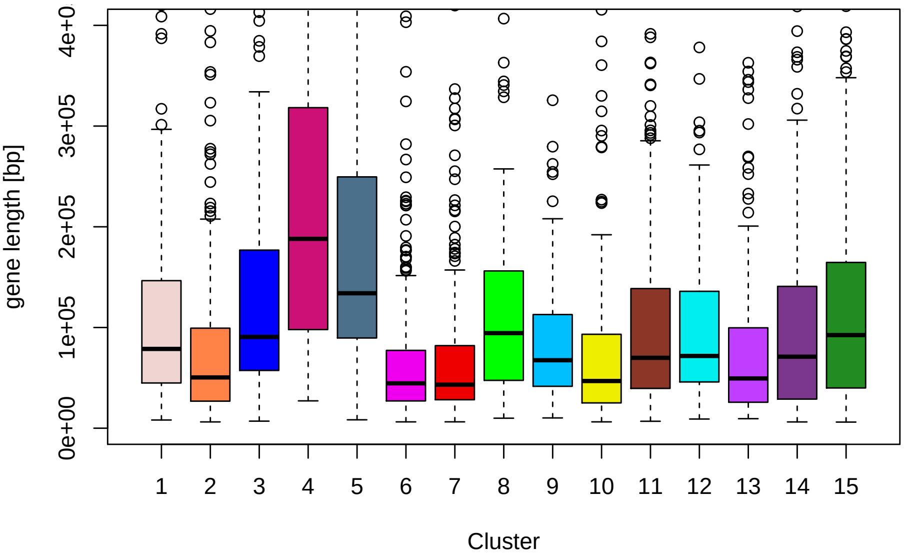

Supplementary Fig. S6 Boxplots showing the distribution of gene lengths for the clusters shown in Fig. 4.

.

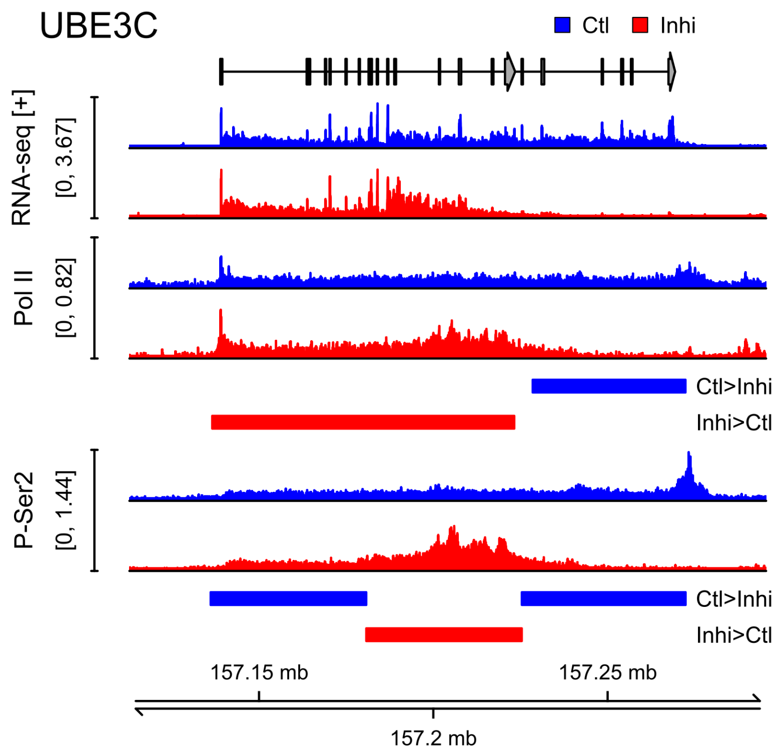

(a)

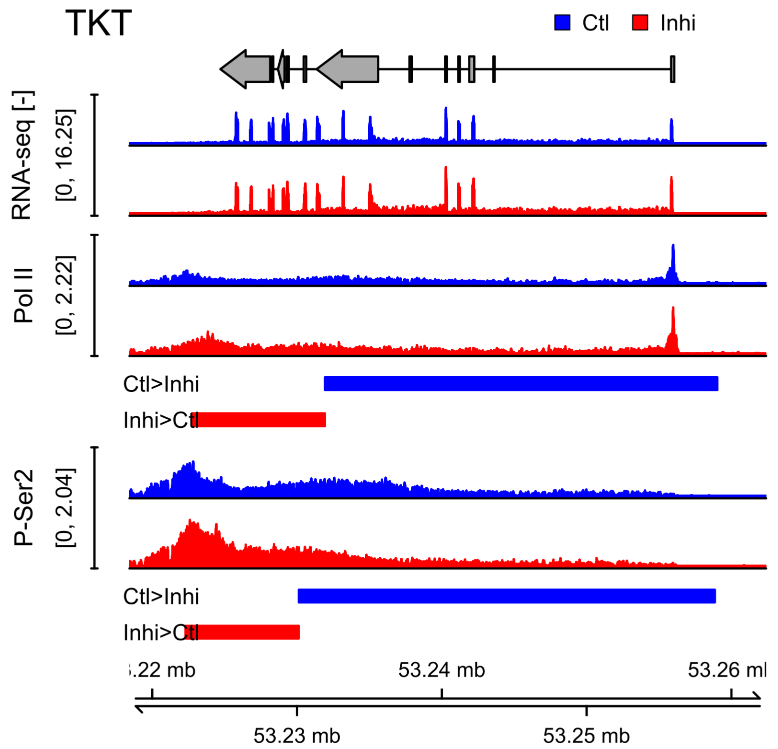

(b)

Supplementary Fig. S7 Read coverage plots showing nuclear RNA-seq data on the respective strand and Pol II and P-Ser2 ChIP-seq data for example genes ((a) UBE3C, from cluster 1 in Fig. 4, (b) TKT, from cluster 15 in Fig. 4) for control (Ctl, blue) and CDK12 inhibitor treatment (Inhi, red). Read counts were normalized to the total number of mapped reads per sample and averaged between replicates. Blue and red boxes below Pol II and P-Ser2 tracks indicate identified regions of change in Pol II and P-Ser2 ChIP-seq data, respectively. Exon (boxes) and intron (lines) structure of corresponding genes is shown on top of subfigures, with gene strand indicated by arrowheads.

4 Comparison to competing approaches

4.1 Comparison to XCAVATOR

Since tools for discovery of copy number variants (CNVs) based on identifying changes in read depth (RD) appear to be potentially useful for identifying regions with differential read distributions, we first evaluated XCAVATOR [Magi et al., 2017] for our applications. XCAVATOR is an RD-based approach that performs differential CNV detection between two conditions (instead of one condition against a reference genome) and is able to also detect multiple copy amplifications. Thus, it appeared most appropriate for identifying genomic regions with differential read distributions among the CNV tools we researched.

Unfortunately, application of XCAVATOR to both PRO-seq data for mock and WT HSV-1 infection from the study by Birkenheuer et al. [2018] and ChIP-seq data for Pol II and P-Ser2 with DMSO (Ctl) or 3-MB-PP1 (Inhi) treatment for 4.5 h from the study by Chirackal Manavalan et al. [2019] did not identify any differential regions. To test that we ran XCAVATOR correctly, we applied it also to low-coverage whole genome shotgun read data from the 1000 genomes project (for individuals NA12878 and NA12815) [1000 Genomes Project Consortium, 2015]. This test did identify ∼17,000 differential regions, which excludes usage errors on our side. The likely explanation why XCAVATOR does not identify any differential regions on the PRO-seq and ChIP-seq data is that changes observed on these data do not follow the assumptions underlying RD-based tools for detecting CNVs. In brief, RD-based tools generally assume that the number of reads mapping to any region of the reference genome (=read count) follows a Poisson distribution and is proportional to the copy number of this region. Thus, the copy number for a genomic region can be estimated from consecutive windows, which should show similar fold-changes to the reference or between conditions (taking into account noise) if they have the same copy number. Furthermore, sudden shifts between consecutive windows with different copy numbers are expected. In contrast, changes in read counts for consecutive subwindows of the differential regions that RegCFinder aims to identify may in- or decrease (see e.g. Fig. 1c,d) and no sudden shifts are observed at the end of the differential regions. It is thus not surprising that XCAVATOR (and similar tools) cannot recover these types of differential regions, for which they were not developed.

4.2 Comparison to diffReps

We also compared RegCFinder against diffReps [Shen et al., 2013]. diffReps also pursues a sliding window-based approach on the whole genome to identify differential regions similar to RD approaches for CNV detection. However, it first determines the significance of a change within each sliding window separately using a negative binomial distribution. Subsequently, diffReps merges overlapping significant windows and recalculates significance of merged windows. This does not require (approximately) the same fold-changes for all subwindows of a differential region and no sudden shifts at the end of regions.

We applied diffReps to both the PRO-seq data and ChIP-seq data using default parameters (in particular, window size = 1000, step size = 100). For this purpose, sequence alignments in BAM format were first converted to the BED format required by diffReps using the bamtobed utility of BEDTools [Quinlan and Hall, 2010]. Since diffReps uses a sliding window approach across the complete genome, no target windows can be defined. To compare diffReps results against RegCFinder, we then analyzed the overlap of significant regions identified by diffReps to input windows used for RegCFinder. Furthermore, since diffReps was developed for ChIP-seq data, it does not consider read strand. Thus, the diffReps analysis for the PRO-seq data was performed in an unstranded manner.

4.3 Results on PRO-seq data for HSV-1 infection

Application of diffReps to PRO-seq data for mock and WT HSV-1 infection identified a total of 71038 differential regions, with 30022 of these determined as up-regulated by diffReps in WT infection and 41016 as down-regulated. Almost all of these (70299 = 99%) were significant at an adjusted p-value cutoff of 0.01 (p-value calculated by diffReps). To directly compare results against RegCFinder, we evaluated the 9040 differential regions identified by diffReps that overlapped the 7650 promoter windows used as input for RegCFinder. Here, 6462 of the promoter windows overlapped with at least one differential region identified by diffReps. Location of these differential windows is visualized in the heatmap in Supplementary Fig. S8 on the left side in the same way as for the RegCFinder results in Fig. 3a . The right side of Supplementary Fig. S8 shows regions identified by RegCFinder for these windows. Here, "WT" (blue) indicates regions found to be up-regulated in HSV-1 infection by diffReps or WT>mock

regions from RegCFinder and "mock" (red) down-regulated regions identified by diffReps or mock>WT regions from RegCFinder. To identify distinct patterns in the regions identified by diffReps, we clustered the heatmap for diffReps results according to Euclidean distances and Ward's clustering criterion. The RegCFinder regions in Supplementary Fig. S8 are ordered according the clustering on the diffReps regions. This comparison showed that, with the exception of (parts of clusters) 7-10, the patterns of changes identified by diffReps differed strongly from the changes identified by RegCFinder. If not noted explicitly otherwise, differential regions mentioned below are differential regions identified by diffReps.

Here, clusters 1-5 represented promoter windows containing almost only differential regions down-regulated in WT compared to mock. For most of these windows (clusters 1, 2, 4 and 5), these regions covered the TSS and upstream (in case of clusters 2 and 5) or downstream regions (in case of clusters 4 and 5). Birkenheuer et al. previously showed using these PRO-seq data that HSV-1 infection leads to a loss of Pol II both at gene promoters and gene bodies for the majority of human genes [Birkenheuer et al., 2018]. This suggests that diffReps identified reduced presence of Pol II at promoters in clusters 1, 2, 4 and 5 as well as gene bodies in clusters 4 and 5, which was confirmed by manual inspection in a genome viewer for example genes from these clusters (Supplementary Fig. S9a,b). Here, diffReps did not identify the relative increases downstream of the TSS in HSV-1 infection found by RegCFinder for many of these genes.

For diffReps clusters 2 and 5, for which down-regulated regions were also found upstream of TSS, inspection indicated that this represented a reduction in antisense transcription from these promoters (Supplementary Fig. S9c) or other close-by promoters on the opposite strand (Supplementary Fig. S9d). Such pairs of close-by promoters on opposite strands with transcription diverging from these promoters are denoted as divergent promoters. Notably, for most human promoters, transcription initiation is

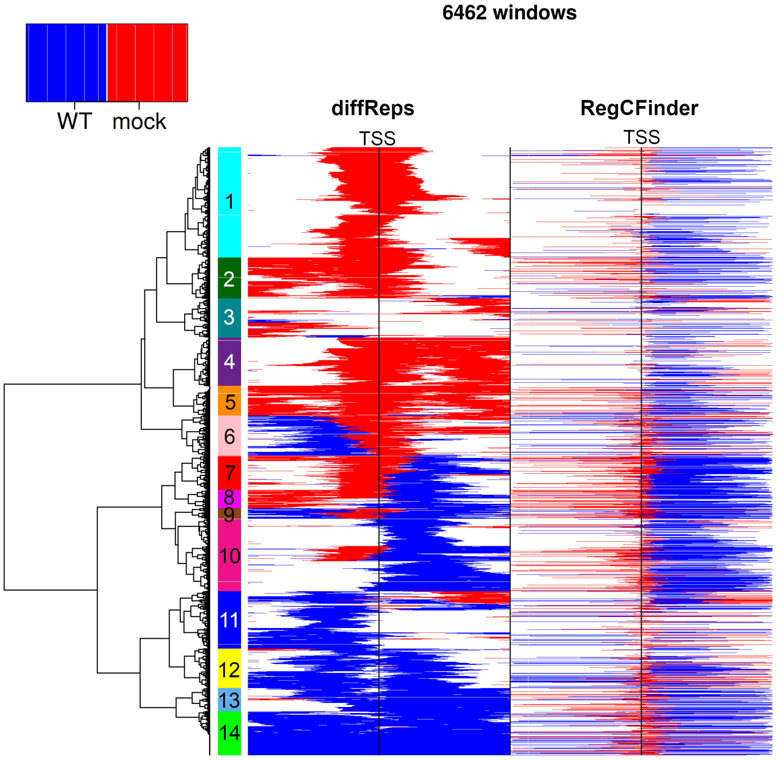

Supplementary Fig. S8 Heatmap showing the location and type of differential regions identified by diffReps (left side) or RegCFinder (right side) for 6462 windows with at least one differential region determined by diffReps (adjusted p-value ≤ 0.01). The TSS is indicated by black vertical lines in both cases. Windows were clustered according to the location of diffReps differential regions and RegCFinder results are shown according to this order (Color scheme: red = regions down-regulated according to diffReps or mock>WT regions according to RegCFinder, blue=regions up-regulated according to diffReps or WT>mock regions according to RegCFinder).

bidirectional but elongation occurs only in the sense direction while antisense transcription is quickly terminated [He et al., 2008, Preker et al., 2008, Seila et al., 2008, 2009]. Reduced levels of Pol II at human promoters during HSV-1 infection thus likely also lead to reduced antisense transcription. While PRO-seq data is strand-specific, diffReps does not consider strand as noted above and thus cannot distinguish between changes in sense and antisense transcription. In summary, diffReps clusters 1-5 predominantly reflect the loss of Pol II on host genes and thus changes in absolute levels of Pol II, but not differences in the distribution of Pol II.

Clusters 6, 9, 11-14 represent windows for which regions up-regulated in HSV-1 infection are found upstream of the TSS by diffReps. Analysis of read-in transcription (Supplementary Fig. S9e) and manual inspection of example genes indicated that up-regulated regions upstream of the TSS partly resulted from either (i) read-in transcription from upstream genes (in particular for some windows in clusters 12 and 14, example in Supplementary Fig. S10a), (ii) divergent promoters (Supplementary Fig. S10b) or (iii) promoters for which (non-productive) antisense transcription continued further into upstream regions before it was terminated (Supplementary Fig. S10c). Consistent with (i), RegCFinder also identified WT>mock regions upstream of the TSS for some windows in diffReps clusters 6, 11, 12 and 14. However, enrichment of read-in transcription for any diffReps clusters was by far not as high as for RegCFinder clusters 3 and 4 (see Fig. 3c), for which a substantial fraction of genes showed read-in >5%, i.e. greater than the cutoff we previously used to distinguish genes with read-in transcription. Thus, RegCFinder clusters 3 and 4 more specifically identify genes with read-in transcription than any of the clusters from the diffReps analysis.

Observations (ii) and (iii) are consistent both with the broadening of antisense Pol II pausing peaks we previously reported [Weiß et al., 2023] and previously reported activation of antisense transcription in HSV-1 infection [Wyler et al., 2017]. Both phenomena lead to increased transcription further upstream of the TSS. However, up-regulated upstream regions were not consistently identified by diffReps for all genes with extended antisense transcription. They were also not identified by RegCFinder, as it performs the analysis in a strand-specific manner. To identify changes in antisense Pol II distribution, input windows for the opposite strand would have to be used.

Up-regulated regions downstream of the TSS observed in clusters 7-10 and 12-14 represented increased Pol II levels on the gene body. Generally, this was either due to (i) increased elongation along the whole gene and potentially downstream of the gene due to read-through transcription beyond poly(A) sites (example in Supplementary Fig. S10c), (ii) a downstream shift in Pol II pausing (Supplementary Fig. S10d) or in a few cases (iii) read-through transcription from a downstream gene on the opposite strand (Supplementary Fig. S10e). However, (i) and (ii) were also observed for windows for which diffReps only identified down-regulated regions (clusters 1, 2, 4 and 5, e.g. Supplementary Fig. S9a). In contrast, RegCFinder often identified WT>mock regions for genes in these clusters.

Accordingly, metagene plots for all diffReps clusters showed a downstream broadening of the PRO-seq signal and differed only in the extent of broadening (Supplementary Fig. S11). Furthermore, consistent with presence of read-in transcription for some genes in clusters 12 and 14, some small increases upstream of the TSS were observed in metagene plots for these clusters (Supplementary Fig. S11d,e). In contrast, metagene plots for RegCFinder clusters showed more diverse patterns, including not only a downstream broadening of PRO-seq peaks but increased upstream levels for RegCFinder clusters 3 and 4 (Supplementary Fig. S3d,e), reflecting the read-in transcription observed for many genes of these clusters, and increased downstream peaks for clusters 7, 8 and 10 (Supplementary Fig. S3a-c) Finally, up-regulated regions identified by diffReps often included the major Pol II peak at the TSS, which was reduced relative to downstream regions (Supplementary Fig. S10b-d,f). In summary, these observations suggest that diffReps predominantly does not detect the change in read distributions but rather absolute changes in read depth.

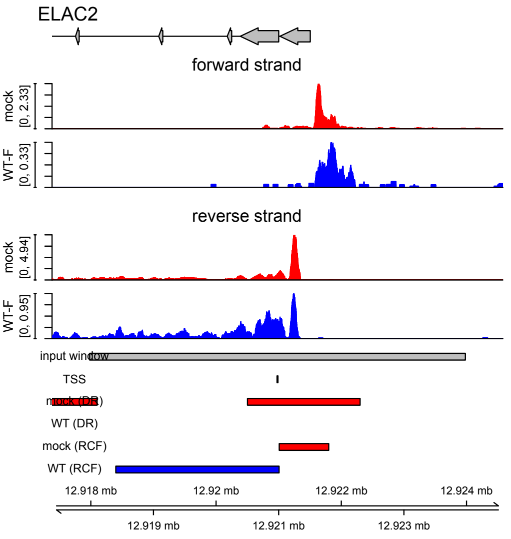

(a)

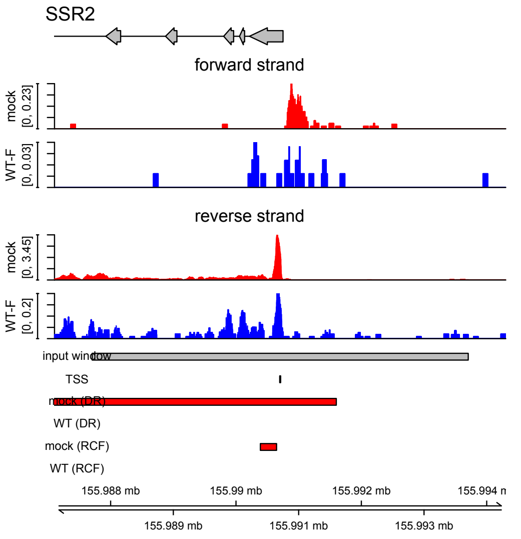

(b)

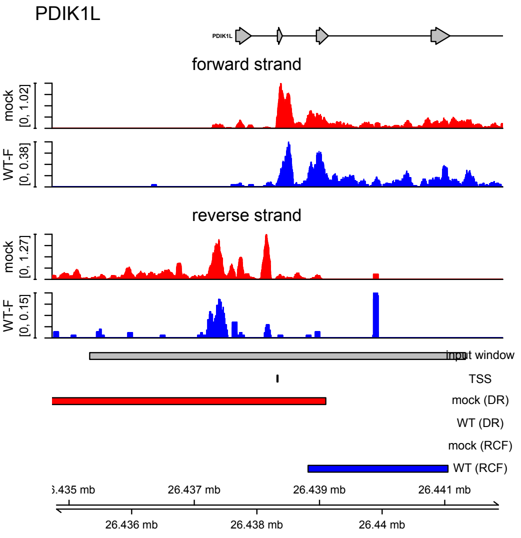

(c)

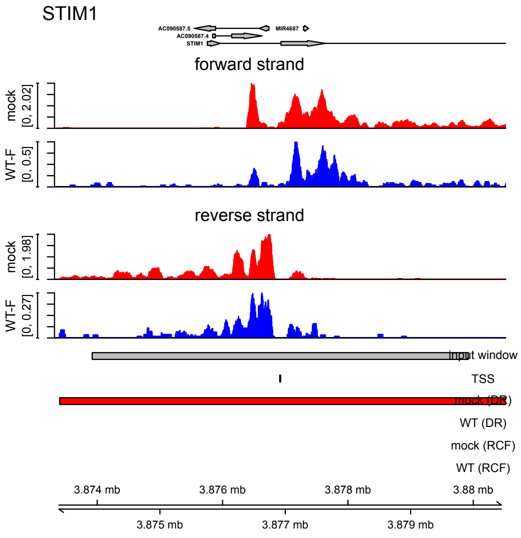

(d)

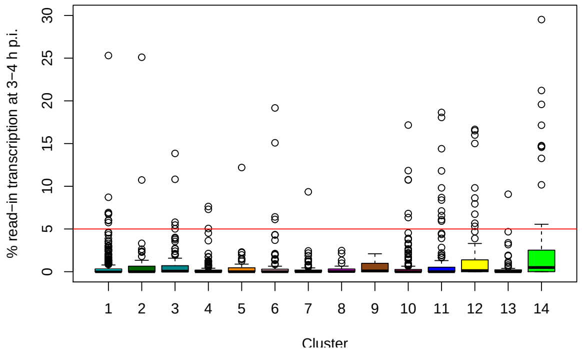

(e)

Supplementary Fig. S9 (a-d) Read coverage plots showing PRO-seq coverage in mock (red) and WT HSV-1 infection (blue) separately on the positive and negative strand for example genes. Input windows (gray), differential regions identified by diffReps (DR, red=down-regulated in HSV-1 infection, blue=up-regulated in HSV-1 infection) and regions of change identified by RegCFinder (RCF, red=mock>WT, blue=WT>mock) are shown below read coverage tracks. Exon (boxes) and intron (lines) structure of genes in this genomic region is shown on top of subfigures, with gene strand indicated by arrowheads. The central gene for which the promoter window was defined is indicated in the top left of subfigures. (e) Boxplots showing the distribution of the % read-in transcription for the 14 clusters obtained for differential regions identified by diffReps in Supplementary Fig. S8. For details on calculation of read-in transcription, see legend to Fig. 3c.

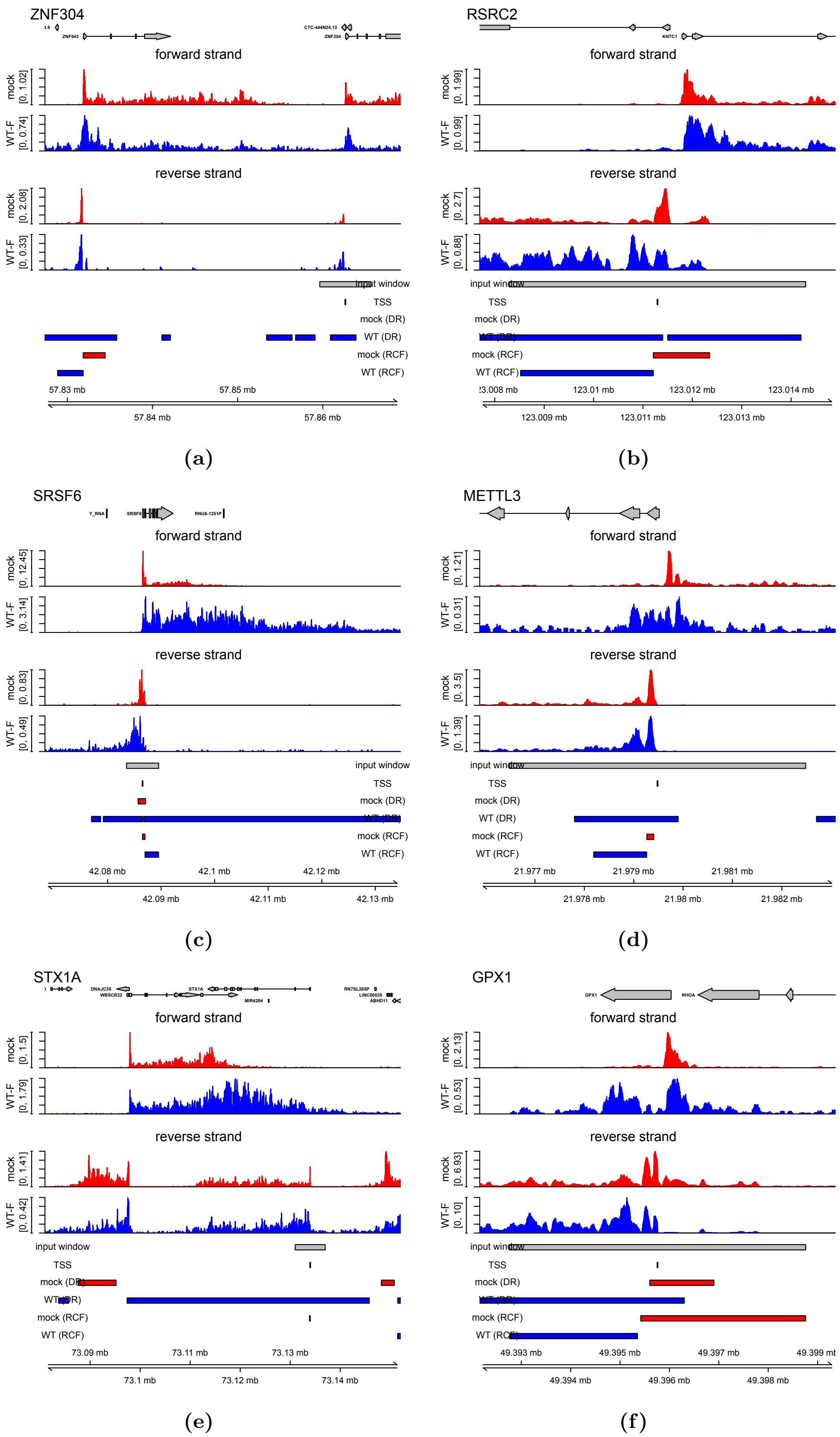

Supplementary Fig. S10 Read coverage plots as in Supplementary Fig. S9 for example genes: (a) example gene (ZNF304, on the right side) with read-in transcription from an upstream gene (ZNF543, on the left side); (b) two genes (RSRC2, KNTC1) with divergent promoters on opposite strands; (c) a gene (SRSF6) with increased elongation along the whole gene and read-through transcription; (d) a gene (METTL3) with a downstream shift in Pol II pausing; (e) a gene (STX1A, reverse strand) for which read-through transcription is observed for a downstream gene on the opposite strand (WBSCR22, forward strand); (f) a gene (GPX1) with a downstream shift in Pol II pausing, for which the up-regulated region identified by diffReps includes the TSS.

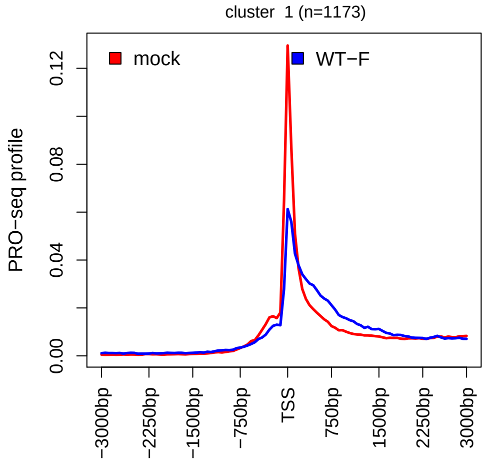

(a)

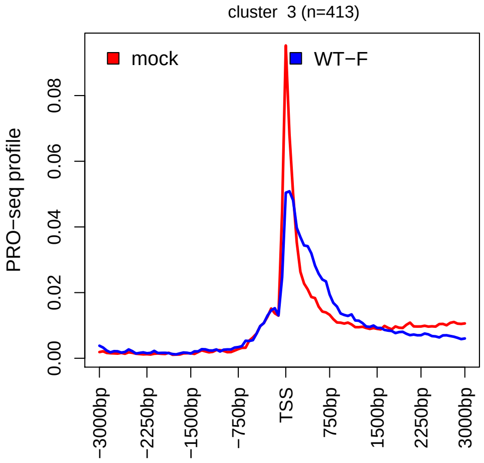

(b)

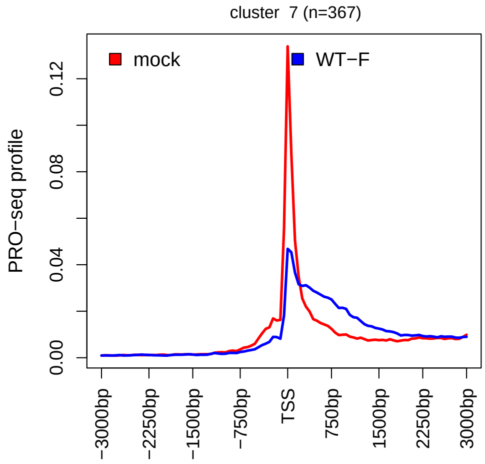

(c)

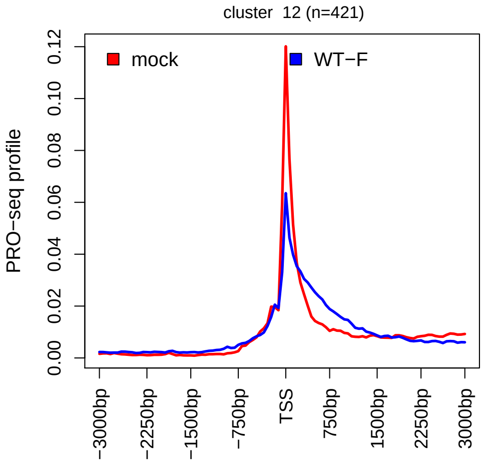

(d)

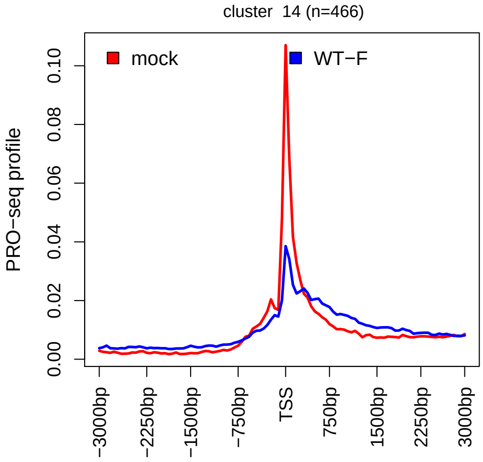

(e)

Supplementary Fig. S11 Metagene curves in the ± 3 kb around the TSS for mock (red) and WT HSV-1 (blue) infection for selected clusters from Supplementary Fig. S8 (cluster numbers and number of genes in each cluster on top of subfigures). For a description on how metagene curves were calculated see caption to Supplementary Fig. S3.

4.4 Results on ChIP-seq data for CDK12 inhibition

Application of diffReps to Pol II and P-Ser2 ChIP-seq data identified 23215 differential regions on the Pol II ChIP-seq data (23004 with adj. p-value < 0.01). 11590 of these were up-regulated and 11625 down-regulated. 10778 ($\sim$46%) of the differential regions overlapped 3630 of the gene windows we had defined as input for RegCFinder (Supplementary Fig. S12a). For the P-Ser2 ChIP-seq data 42064 differential regions were identified (41909 with adj. p-value < 0.01), with 15762 region up-regulated and 26302 down-regulated. 20313 ($\sim$48%) of these differential windows overlapped 4776 of the gene windows (Supplementary Fig. S12b).

Clustering analysis of the location heatmaps indicated that in both cases a large fraction of gene windows (39% and 22% for the Pol II and P-Ser2 ChIP-seq data, respectively) belonged to one large cluster with no specific pattern regarding the location of differential regions. This was even the case when selecting a relatively stringent clustering cutoff for the P-Ser2 data, which resulted in 20 clusters. As the differential regions identified by diffReps on the Pol II ChIP-seq data were fewer and covered smaller fractions of input gene windows, we focused on the diffReps results on the P-Ser2 data for the comparison against RegCFinder. Here, more distinctive patterns were observed and many clusters showed down-regulation at or close to the end of genes upon inhibitor treatment (clusters 1, 4, 5, 8, 11, 12, 14-18 and 20) as expected from our previous study [Chirackal Manavalan et al., 2019]. Some, but not all of these clusters also exhibited regions up-regulated upon inhibitor treatment upstream of these down-regulated regions (clusters 15-20).

Supplementary Fig. S12c shows a direct comparison of differential regions identified by diffReps (left) and RegCFinder (right) on the P-Ser2 data. diffReps identified on average twice as many regions per window (4.25 on average) than RegCFinder (2.16 on average). Often this was due to diffReps identifying multiple small regions where RegCFinder identified one large region covering all of these (see e.g. Supplementary Fig. S13a-e). Thus, some sort of clustering of close-by differential regions from diffReps would have to be applied to group these together into "meta-regions" for downstream analyses.

Apart from the large, non-specific cluster 9, RegCFinder generally identified the same differential regions as diffReps – but commonly as one continuous region rather than the multiple smaller regions identified by diffReps – as well as additional up- and/or downstream regions (e.g. Supplementary Fig. S13b,d,f). Manual inspection of example genes confirmed that in many cases a clear relative increase in reads was observed in these additional regions identified by RegCFinder (e.g. Inhi>Ctl regions in Supplementary Fig. S13b,f). Other additional differential regions reflect a shift in the relative distribution of reads from these regions to other regions in the window (e.g. Ctl>Inhi regions in Supplementary Fig. S13d,e). That diffReps does not identify these regions as differential likely reflects the different objective of diffReps, which focuses on identifying regions with differences in ChIP-seq enrichment rather than changes in the distribution of reads within particular windows.

For the large non-specific cluster 9, diffReps identified only short (relative to gene length) differential regions and no consistent patterns with no similarity to RegCFinder results. diffReps cluster 9 contained very long genes (Supplementary Fig. S14a, e.g. Supplementary Fig. S13g). Consistently, genes from RegCFinder clusters 4 and 5, which represented the clusters with the longest genes in the RegCFinder clustering analysis, were strongly enriched in diffReps cluster 9. Here, diffReps cluster 9 contained around 48% of RegCFinder clusters 4 and 5 genes, but diffReps results did not reflect the distinctive pattern identified by RegCFinder with a relative increase in P-Ser2 close to the gene start upon CDK12 inhibition and a decrease of P-Ser2 further downstream. Thus, the loss of P-Ser2 signal towards gene ends in particular for long genes is not as clearly reflected in diffReps results as in RegCFinder results. In part, this is likely due to the fact that these genes are also among the most lowly expressed genes (according to nuclear RNA-seq data, Supplementary Fig. S14b). Thus, diffReps likely does not identify these differential regions due to low read counts (see e.g. Supplementary Fig. S13h).

In summary, our comparison shows that diffReps identifies only a subset of differential regions with changes in the P-Ser2 distribution identified by RegCFinder on the input gene windows, while RegCFinder recovers most differential regions identified by diffReps.

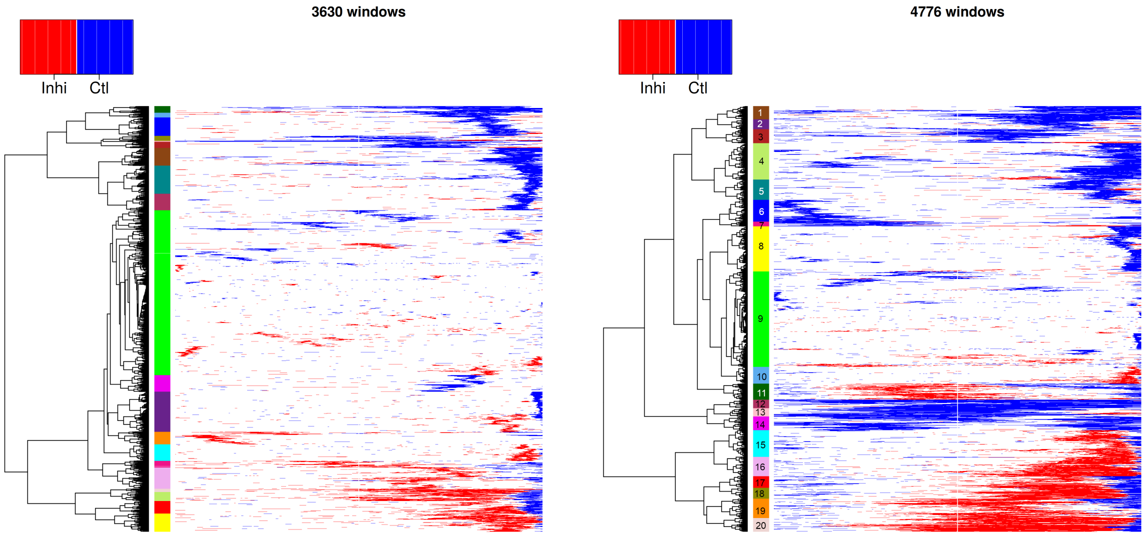

(a) **(b)**

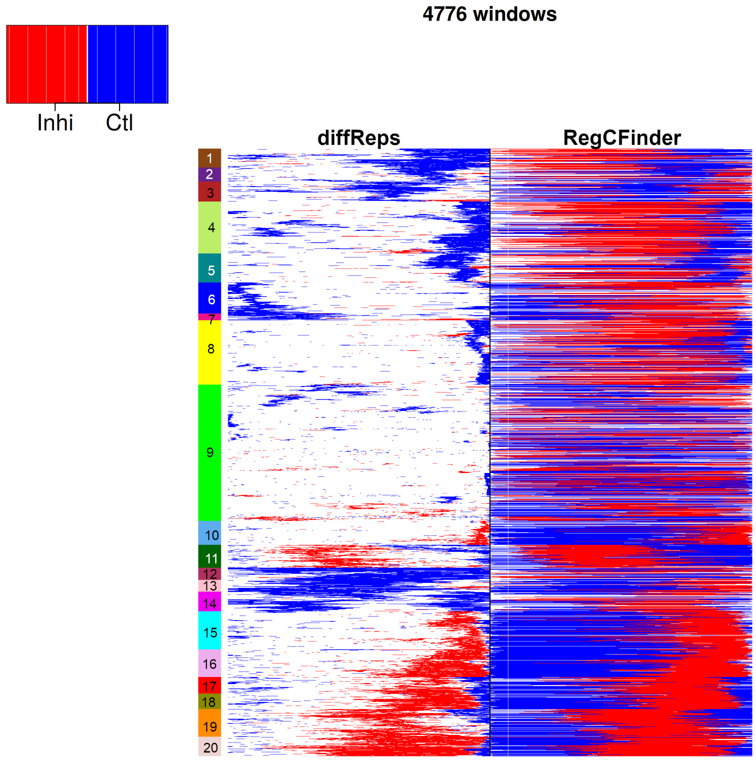

(c)

Supplementary Fig. S12 (a,b) Heatmap showing the location and type of differential regions identified by diffReps on the Pol II (a) and P-Ser2 (b) ChIP-seq data for windows with at least one differential region determined by diffReps (adjusted p-value $\leq$ 0.01). (c) Heatmap showing the location and type of differential regions identified by diffReps (left side) or RegCFinder (right side) for the P-Ser2 ChIP-seq data for windows with at least one differential region determined by diffReps (adjusted p-value $\leq$ 0.01). Windows were ordered according to the clustering in (b). Color scheme: blue = regions down-regulated according to diffReps upon inhibitor treatment or Ctl>Inhi regions according to RegCFinder, red=regions up-regulated according to diffReps or Inhi>Ctl regions according to RegCFinder. Cluster numbers are indicated on the left.

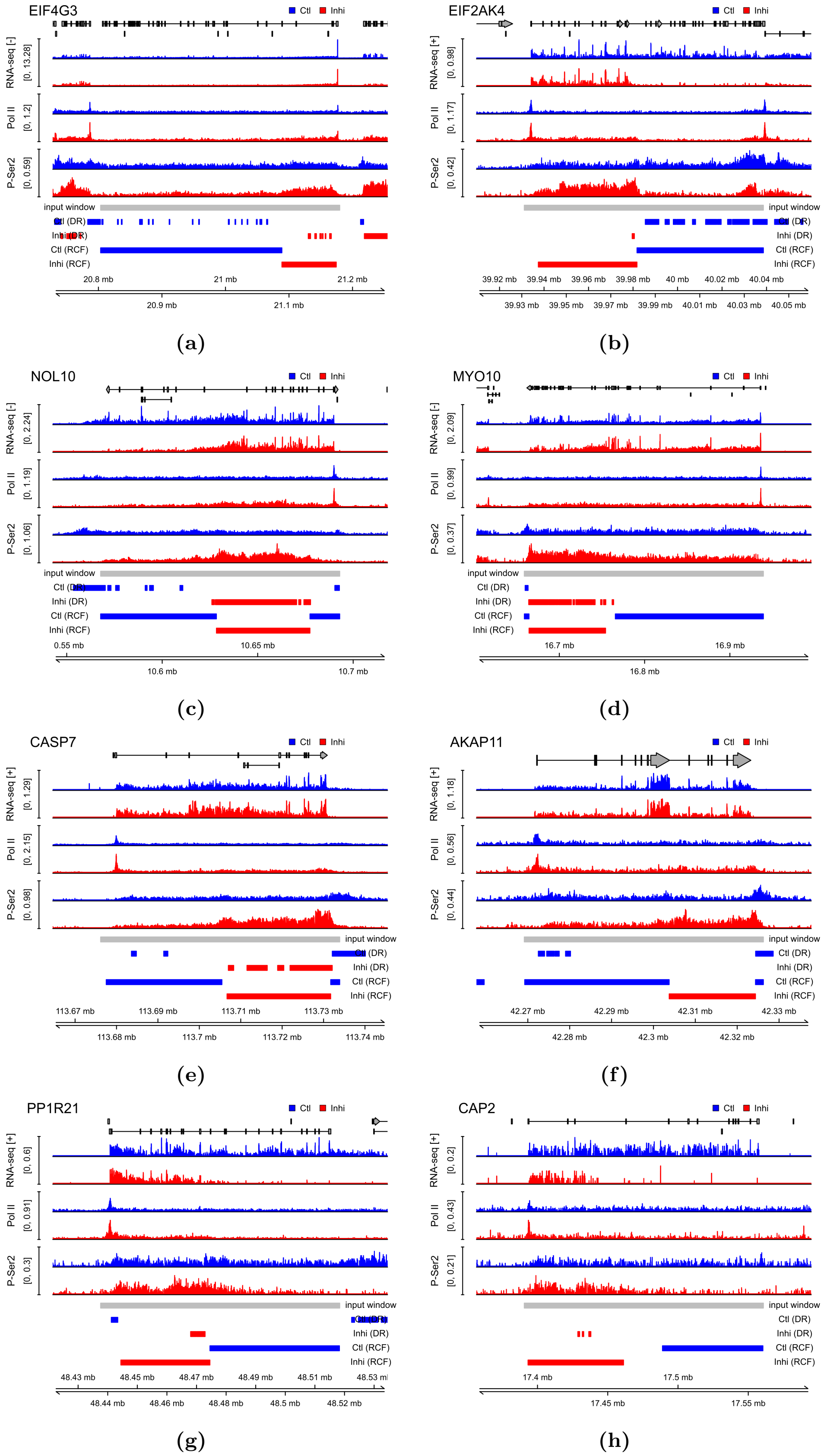

Supplementary Fig. S13 Read coverage plots showing nuclear RNA-seq data on the respective strand and Pol II and P-Ser2 ChIP-seq data for example genes for control (Ctl, blue) and CDK12 inhibitor treatment (Inhi, red). Read counts were normalized to the total number of mapped reads per sample and averaged between replicates. Input windows (gray), differential regions identified by diffReps (DR, blue=down-regulated upon inhibitor treatment, red=up-regulated upon inhibitor treatment) and regions of change identified by RegCFinder (RCF, blue=Ctl>Inhi, red=Inhi>Ctl) are shown below read coverage tracks. Exon (boxes) and intron (lines) structure of corresponding genes is shown on top of subfigures, with gene strand indicated by arrowheads. Gene symbols of the central gene for which the window was defined are shown on the top left.

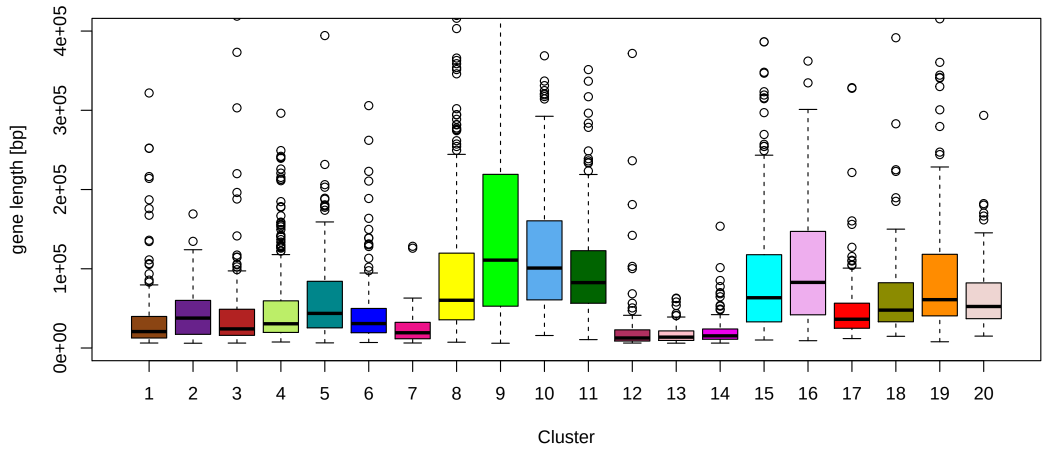

(a)

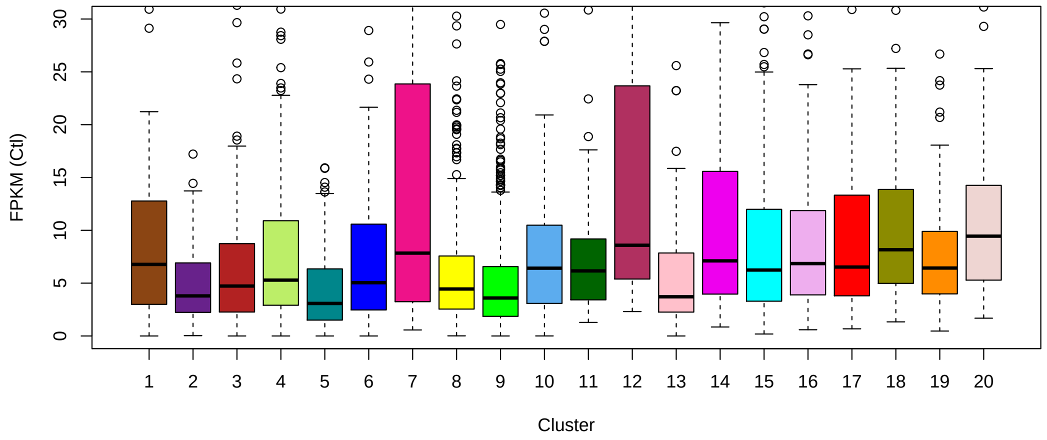

(b)

Supplementary Fig. S14 Boxplots showing distribution of (a) gene length and (b) gene expression (calculated as fragments per million mapped reads =: FPKM) in nuclear RNA-seq data for control samples for the 20 clusters identified from the location heatmap for differential regions identified by diffReps on the P-Ser2 data from Supplementary Fig. S12b.

References

1000 Genomes Project Consortium. A global reference for human genetic variation. *Nature*, 526:68–74, 2015.

C. H. Birkenheuer, C. G. Danko, and J. D. Baines. Herpes simplex virus 1 dramatically alters loading and positioning of RNA Polymerase II on host genes early in infection. *Journal of virology*, 92, Apr. 2018.

T. Bonfert, E. Kirner, G. Csaba, R. Zimmer, and C. C. Friedel. ContextMap 2: fast and accurate context-based RNA-seq mapping. *BMC bioinformatics*, 16:122, Apr. 2015.

A. P. Chirackal Manavalan, K. Pilarova, M. Kluge, K. Bartholomeeusen, M. Rajecky, J. Oppelt, P. Khirsariya, K. Paruch, L. Krejci, C. C. Friedel, and D. Blazek. Cdk12 controls G1/S progression by regulating RNAPII processivity at core DNA replication genes. *EMBO reports*, 20:e47592, Sept. 2019.

P. Danecek, J. K. Bonfield, J. Liddle, J. Marshall, V. Ohan, M. O. Pollard, A. Whitwham, T. Keane, S. A. McCarthy, R. M. Davies, and H. Li. Twelve years of samtools and bcftools. *GigaScience*, 10: giab008, Feb. 2021.

Y. He, B. Vogelstein, V. E. Velculescu, N. Papadopoulos, and K. W. Kinzler. The antisense transcriptomes of human cells. *Science (New York, N.Y.)*, 322:1855–1857, 2008.

C. S. Jürges, L. Dölken, and F. Erhard. Integrative transcription start site identification with iTiSS. *Bioinformatics*, 37:3056–3057, 2021.

H. Li and R. Durbin. Fast and accurate short read alignment with Burrows-Wheeler transform. *Bioinformatics (Oxford, England)*, 25:1754–1760, July 2009.

A. Magi, T. Pippucci, and C. Sidore. XCAVATOR: accurate detection and genotyping of copy number variants from second and third generation whole-genome sequencing experiments. *BMC genomics*, 18: 747, 2017.

P. Preker, J. Nielsen, S. Kammler, S. Lykke-Andersen, M. S. Christensen, C. K. Mapendano, M. H. Schierup, and T. H. Jensen. RNA exosome depletion reveals transcription upstream of active human promoters. *Science*, 322:1851–1854, 2008.

A. R. Quinlan and I. M. Hall. BEDTools: a flexible suite of utilities for comparing genomic features. *Bioinformatics*, 26:841–842, 2010.

A. C. Seila, J. M. Calabrese, S. S. Levine, G. W. Yeo, P. B. Rahl, R. A. Flynn, R. A. Young, and P. A. Sharp. Divergent transcription from active promoters. *Science*, 322:1849–1851, 2008.

A. C. Seila, L. J. Core, J. T. Lis, and P. A. Sharp. Divergent transcription: a new feature of active promoters. *Cell cycle*, 8:2557–2564, 2009. ISSN 1551-4005. doi: 10.4161/cc.8.16.9305.

L. Shen, N.-Y. Shao, X. Liu, I. Maze, J. Feng, and E. J. Nestler. diffReps: detecting differential chromatin modification sites from ChIP-seq data with biological replicates. *PloS one*, 8:e65598, 2013.

E. Weiß, T. Hennig, P. Graßl, L. Djakovic, A. W. Whisnant, C. S. Jürges, F. Koller, M. Kluge, F. Erhard, L. Dölken, and C. C. Friedel. HSV-1 infection induces a downstream shift of promoter-proximal pausing for host genes. *Journal of virology*, page e0038123, 2023.

E. Wyler, J. Menegatti, V. Franke, C. Kocks, A. Boltengagen, T. Hennig, K. Theil, A. Rutkowski, C. Ferrai, L. Baer, L. Kermas, C. Friedel, N. Rajewsky, A. Akalin, L. Dölken, F. Grässer, and M. Landthaler. Widespread activation of antisense transcription of the host genome during herpes simplex virus 1 infection. *Genome biology*, 18:209, 2017.
